# Nitroimidazole carboxamides as antiparasitic agents targeting *Giardia lamblia*, *Entamoeba histolytica* and *Trichomonas vaginalis*

**DOI:** 10.1016/j.ejmech.2016.04.064

**Published:** 2016-09-14

**Authors:** A.M. Jarrad, A. Debnath, Y. Miyamoto, K.A. Hansford, R. Pelingon, M.S. Butler, T. Bains, T. Karoli, M.A.T. Blaskovich, L. Eckmann, M.A. Cooper

**Affiliations:** a Institute for Molecular Bioscience, The University of Queensland, Brisbane, Queensland, 4072, Australia; b Center for Discovery and Innovation in Parasitic Diseases, Skaggs School of Pharmacy and Pharmaceutical Sciences, University of California, San Diego, La Jolla, CA, USA; c Department of Medicine, University of California, San Diego, La Jolla, CA, USA

**Keywords:** Nitroimidazole, Antiparasitic agent, *Giardia lamblia*, *Entamoeba histolytica*, Plasma protein binding, Metabolism, MtzS, metronidazole sensitive, MtzR, metronidazole resistant, MIC, minimum inhibition concentration

## Abstract

Diarrhoeal diseases caused by the intestinal parasites *Giardia lamblia* and *Entamoeba histolytica* constitute a major global health burden. Nitroimidazoles are first-line drugs for the treatment of giardiasis and amebiasis, with metronidazole **1** being the most commonly used drug worldwide. However, treatment failures in giardiasis occur in up to 20% of cases and development of resistance to metronidazole is of concern. We have re-examined ‘old’ nitroimidazoles as a foundation for the systematic development of next-generation derivatives. Using this approach, derivatisation of the nitroimidazole carboxamide scaffold provided improved antiparasitic agents. Thirty-three novel nitroimidazole carboxamides were synthesised and evaluated for activity against *G. lamblia* and *E. histolytica*. Several of the new compounds exhibited potent activity against *G. lamblia* strains, including metronidazole-resistant strains of *G. lamblia* (EC_50_ = 0.1–2.5 μM cf. metronidazole EC_50_ = 6.1–18 μM). Other compounds showed improved activity against *E. histolytica* (EC_50_ = 1.7–5.1 μM cf. metronidazole EC_50_ = 5.0 μM), potent activity against *Trichomonas vaginalis* (EC_50_ = 0.6–1.4 μM cf. metronidazole EC_50_ = 0.8 μM) and moderate activity against the intestinal bacterial pathogen *Clostridium difficile* (0.5–2 μg/mL, cf. metronidazole = 0.5 μg/mL). The new compounds had low toxicity against mammalian kidney and liver cells (CC_50_ > 100 μM), and selected antiparasitic hits were assessed for human plasma protein binding and metabolic stability in liver microsomes to demonstrate their therapeutic potential.

## Highlights

• A series of 5-, 4(5)- and 4-nitroimidazoles were synthesised.
• SAR against protozoan parasites was established.
• Several compounds were more potent than metronidazole against *G. lamblia* and *E. histolytica*
• Most compounds were not cytotoxic at 100 μM and were stable to microsomal metabolism.
• Rediscovery of ‘old’ nitroimidazoles can identify agents with therapeutic potential.

## Graphical abstract

**Figure:**
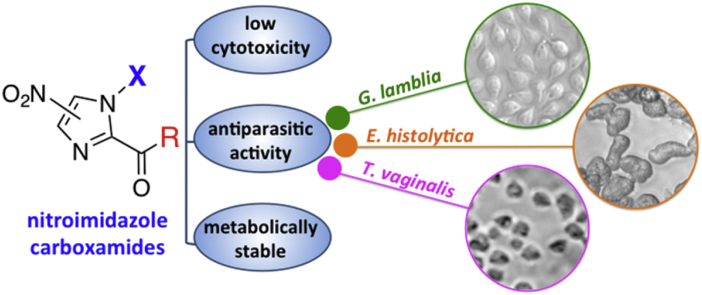

## 1 Introduction

Diarrhoeal diseases caused by intestinal protozoan parasites are a major global health burden. Two of the most common intestinal parasites, *Giardia lamblia* and *Entamoeba histolytica*, are responsible for ∼280 million and ∼50 million annual infections, respectively [1], [2]. Transmission of these parasites occurs by the faecal-oral route through ingestion of cysts in contaminated water or food, or by direct person-to-person contact. *G. lamblia* may also have animal reservoirs, making the infection a potential zoonotic disease [3]. Upon ingestion of *G. lamblia* cysts, trophozoites emerge from the cysts and multiply in the lumen of the small intestine, where they can attach to the intestinal mucosa. Symptoms of acute giardiasis include watery diarrhoea, abdominal discomfort, pain and cramps. Chronic disease can result in malabsorption and failure to thrive in children [4]. For amebiasis, trophozoites migrate to the large intestine and can either reside in the lumen or invade the colonic mucosa or other extra-intestinal sites, most prominently the liver [5].

Due to the propensity for spread through contaminated water and food sources and the low infectious dose of *G. lamblia* and *E. histolytica* cysts [6], the global disease burden is disproportionately shouldered by developing nations in areas with inadequate sanitation. Protozoan diseases also impact developed nations, often via travellers visiting regions where disease is endemic. The threat to developed nations is recognised by the US National Institute of Allergy and Infectious Diseases as both protozoa are category B bioterrorism threat pathogens [7].

**Fig. 1:**
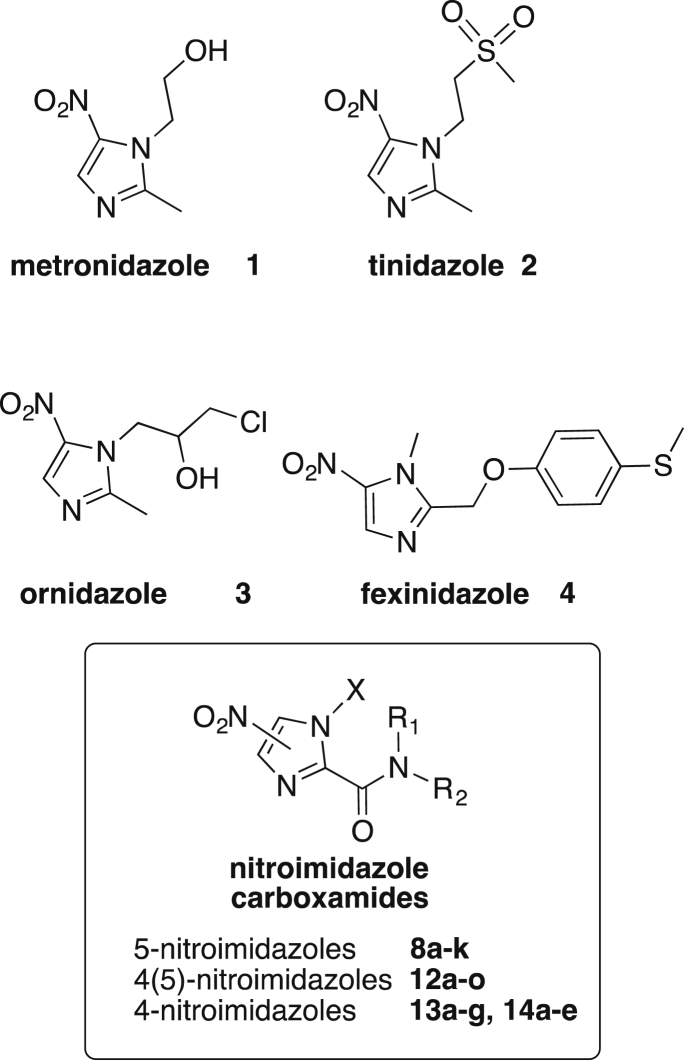
5-Nitroimidazoles **1–4** in clinical use or development for treatment of parasitic diseases, while **8a-k**, **12a-o**, **13a-g** and **14a-e** are the nitroimidazole carboxamides investigated here.

Metronidazole **1** (Fig. 1) is a generic drug for treatment of a range of parasitic and anaerobic bacterial infections. For giardiasis, metronidazole is typically given in 250 mg doses three times a day for 5–7 days, while amebiasis is treated with a higher 750 mg dose three times a day for 5–10 days, often followed by treatment with paromomycin to eradicate cysts from the colon [8], [9]. Other 5-nitroimidazoles, such as tinidazole **2** and ornidazole **3** (Fig. 1) have improved dosing schedules with only a single 2 g tablet of either drug for treatment of giardiasis, or 2 g tinidazole once daily for three days for treatment of amebiasis [8], [9], [10]. These agents have similar adverse effects such as nausea, vomiting and headaches. Ornidazole is not approved for use in the United States [8], [9], [10]. Unfortunately, metronidazole treatment fails in up to 20% of giardiasis cases with metronidazole resistance an ever increasing concern [11], [12]. Parasites resistant to metronidazole show cross-resistance to tinidazole [1]. Furthermore, resistance of *E. histolytica* to metronidazole has also been described, as trophozoites can be adapted to grow in the presence of therapeutically relevant levels of metronidazole [13]. Given the sheer number of cases of giardiasis and amebiasis, and treatment failures, development of alternative treatment options remains an important priority.

Re-examination of ‘old’ nitroimidazoles is a valuable strategy in the development of new drugs for treatment of parasitic diseases. For example, fexinidazole **4** (Fig. 1), initially discovered in the 1980s, has been “rediscovered” and is in clinical development by the Drugs for Neglected Diseases initiative for treatment of Human African trypanosomiasis (sleeping sickness) and Chagas disease [14]. Metronidazole has been in clinical use for over 50 years, but the expanded potential of metronidazole based agents has recently been demonstrated by modifying metronidazole with a “click chemistry” approach to generate agents with improved potency and activity against metronidazole resistant (MtzR) parasites [15], [16].

Nitroimidazole carboxamides (Fig. 1) were originally patented by Merck &. Co. in 1973 for the treatment of infections caused by *Histomonas meleagridis* and *Trichomonas vaginalis* [17]. *H. meleagridis* is a parasite that causes lesions in the cecum and liver of chickens and turkeys, and is commonly known as turkey blackhead disease [18]. In contrast, *T. vaginalis* infects the genitourinary tract in humans causing inflammation and vaginal discharge in women [19]. The nitroimidazole carboxamides displayed efficacy in *in vivo* turkey and mouse models of *Histomonas maleagridis* and *T. vaginalis* [17], respectively, but no substantial antimicrobial development of this series has since been reported. Given the core 5-nitroimidazole group in the nitroimidazole carboxamides is similar to metronidazole, we hypothesised that these compounds could have therapeutic potential against enteric parasites, including *G. lamblia* and *E. histolytica*. In addition, the 2′-carboxamide substitution provides a convenient handle to optimise antiparasitic properties. Therefore, we explored the structure activity relationships (SAR) of nitroimidazole carboxamides and conducted preliminary ADME studies to identify improved antiparasitic agents with therapeutic potential against *G. lamblia* and *E. histolytica*.

## 2 Materials and methods

### 2.1 Synthesis of 1-methyl-5-nitroimidazoles

**Scheme 1:**
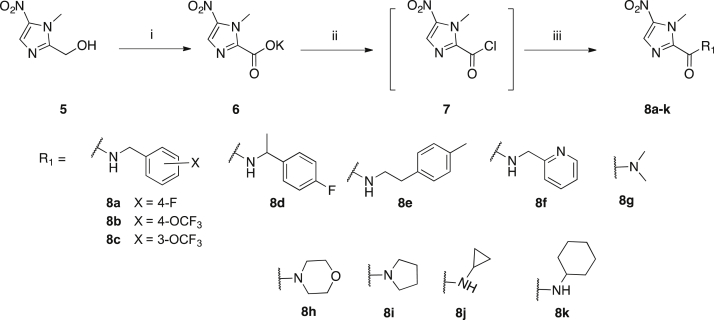
Synthesis of 1-methyl-5-nitroimidazoles **8a-k**. i) KMnO_4_, acetone, −5 °C → rt, 85%; ii) oxalyl chloride, cat. DMF, DCM, 0 °C → rt; iii) amine, TEA, DCM, 0 °C → rt, 19–58%.

The library of 1-methyl-5-nitroimidazole carboxamides **8a-k** was prepared essentially as described by Hoff [17] using the synthetic approach depicted in Scheme 1. Commercially available 1-methyl-2-hydroxymethyl-5-nitro imidazole **5** was oxidised with potassium permanganate in acetone to form the potassium carboxylate salt **6**, which was isolated in this form to avoid decarboxylation of the free carboxylic acid [17]. The crude carboxylate salt **6**, upon reaction with oxalyl chloride and catalytic DMF, provided the acid chloride intermediate **7**. The library of 5-nitroimidazole carboxamides **8a-k** was then prepared by reacting the crude acid chloride **7** with the desired primary or secondary amines in the presence of triethylamine (Scheme 1). Compounds **8a-f** and **8i-k** are first reported here. Compounds **8g** (R = NMe_2_) and **8h** (R = morpholine), originally reported by Hoff [17], were prepared for use as comparators due to their activity in *in vivo* models of *T. vaginalis* and *H. meleagridis* infection. The title compounds **8a-k** were all purified by direct or reverse phase chromatography to ≥95% purity before biological testing. All compounds were characterised by ^1^H and ^13^C NMR, LCMS and HRMS and detailed experimental procedures and characterisation data are provided in the supplementary information.

### 2.2 Synthesis of 4(5)-nitroimidazoles

**Scheme 2:**
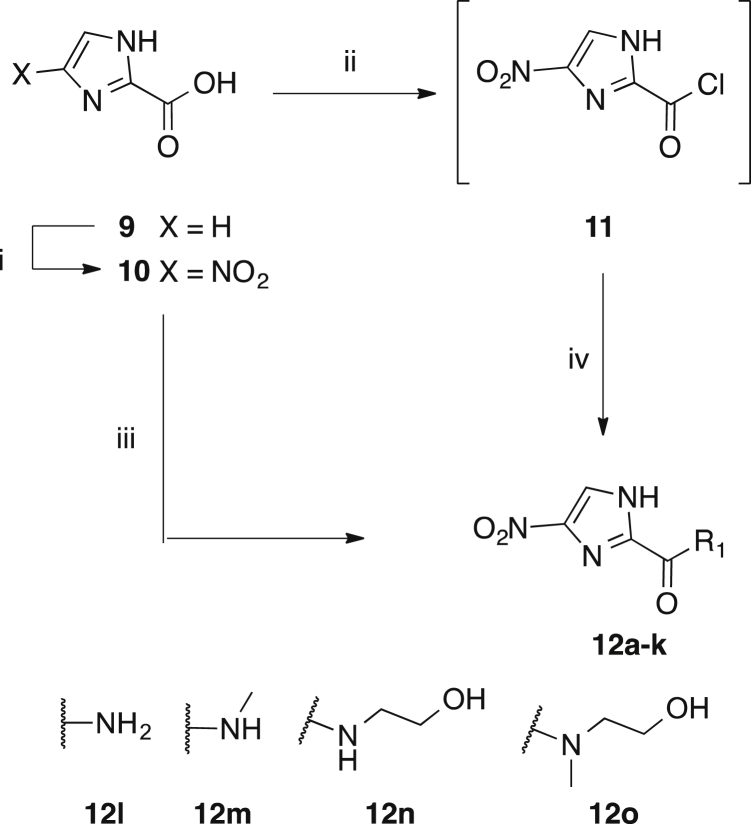
Synthesis of 4(5)-nitroimidazoles **12a-o**. Amide groups **a-k** are as defined in Scheme 1. i) HNO_3_, H_2_SO_4_, 80 °C, 54%; ii) oxalyl chloride, cat. DMF, DCM, 0 °C → rt; iii) amine, PyBOP, DIPEA, 6–75% iv) amine, TEA, DCM, 0 °C → rt, 12–93%.

To examine the corresponding 4(5)-nitroimidazole carboxamide series of **8a-k** (i.e no *N*-methyl substitution) we prepared the analogous series of novel compounds **12a-k**. In addition, four alternative novel carboxamides **12l-o** were prepared, as shown in Scheme 2. Imidazole-2-carboxylic acid **9** was readily nitrated with conc. HNO_3_/H_2_SO_4_ to give 4(5)-nitroimidazole carboxylic acid **10**. Carboxamides **12a-o** were subsequently prepared by activation of acid **10** (oxalyl chloride/catalytic DMF or PyBOP/DIPEA) followed by coupling of the requisite amine. Amidation via intermediate **11** was the preferred route due to the difficulty of removing the HOBt and tripyrrolidinophosphine oxide by-products formed during the PyBOP mediated coupling. The primary amide **12l** was prepared by quenching the acid chloride **11** with concentrated ammonium hydroxide solution. The title compounds **12a-o** were all purified and characterised as described for **8a-k**.

### 2.3 Synthesis of 4-nitroimidazoles

**Scheme 3:**
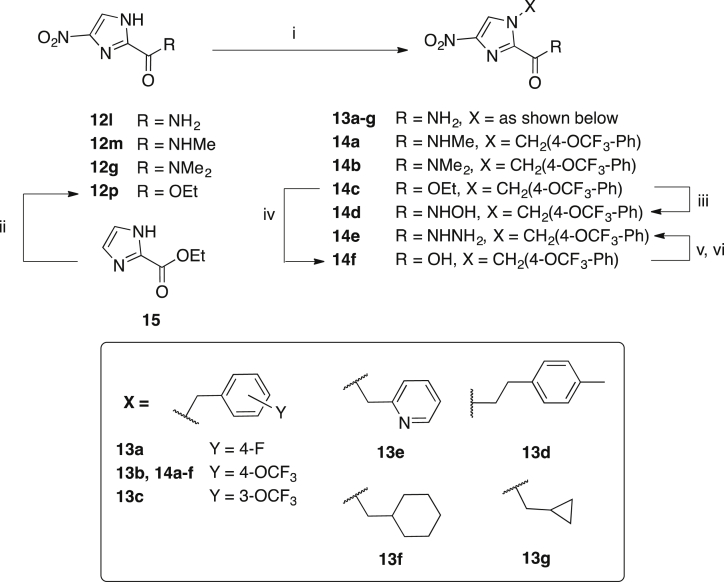
Synthesis of 4-nitroimidazoles **13a-g** and **14a-f**. i) benzyl or alkyl bromide, K_2_CO_3_, DMF, rt → μW 80 °C, 7–98%; ii) HNO_3_, H_2_SO_4_, 60 °C, 64%; iii) NH_2_OH, MeOH, 60 °C, 37%; iv) 1 M NaOH, THF: MeOH (1:1), rt, 37%; v) oxalyl chloride, cat. DMF, DCM, 0 °C → rt; vi) NH_2_NH_2_•H_2_O, DCM, 0 °C, 65%.

The novel 4-nitroimidazoles **13a-g** and **14a-c** were synthesised from the respective 1H-imidazole carboxamides **12g**, **12l, 12m** and **12p** by alkylation with benzyl or alkyl halides under basic conditions (K_2_CO_3_) (Scheme 3). The hydroxamic acid **14d** was prepared by treatment of the ester **14c** with hydroxylamine in methanol at 60 °C. The hydrazide **14e** was prepared from **14f** via an acid chloride intermediate and hydrazine. The title compounds **13a-g** and **14a-e** were purified by chromatography or recrystallisation and characterised as described for **8a-k**.

### 2.4 Synthesis of des-nitroimidazoles and 4(5)-amino-imidazole carboxamide

**Scheme 4:**
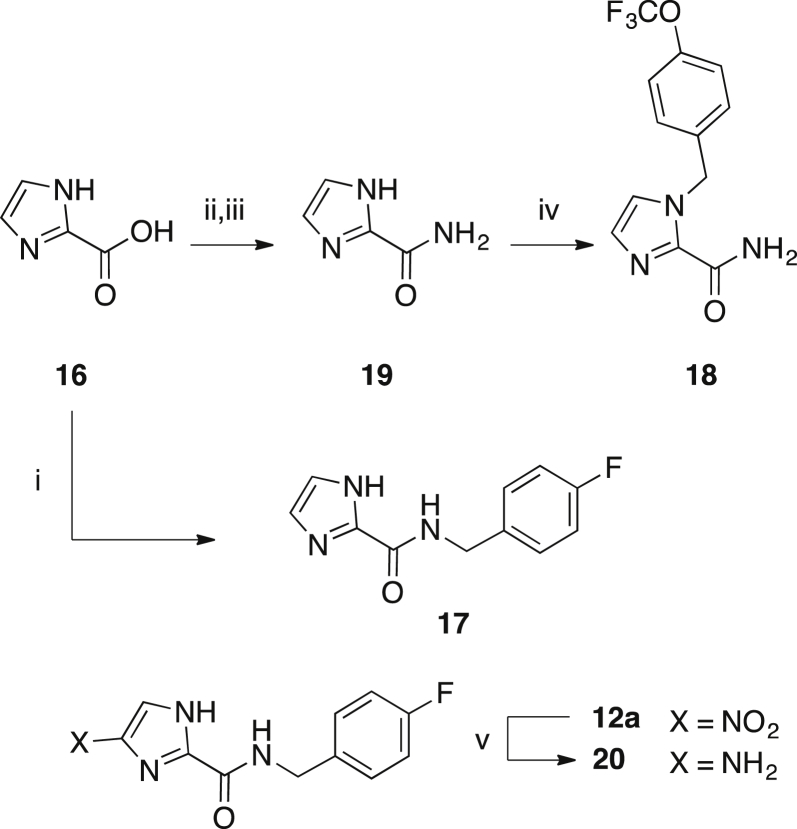
Synthesis of desnitro-imidazoles **17** and **18** and amine **20**. i) 4-fluorobenzylamine, PyBOP, DIPEA, DCM, rt, 67% ii) oxalyl chloride, cat. DMF, 0 °C → rt; iii) conc. NH_4_OH, 0 °C → rt; 73%; iv) 4-(trifluoromethoxy)benzyl bromide, K_2_CO_3_, DMF, rt, 58%; v) MeOH, Pd/C, H-cube, 1 atm, 30 °C, 74%.

Des-nitro imidazole **17** was prepared by coupling **16** and 4-fluorobenzylamine with PyBOP and DIPEA (Scheme 4). The desnitro-imidazole **18** was prepared by alkylation of **19** with 4-(trifluoromethoxy)benzyl bromide in DMF under basic conditions achieved with K_2_CO_3_ (Scheme 4). The 4(5)-nitroimidazole carboxamide **19** was prepared from the intermediate acid chloride formed with oxalyl chloride and catalytic DMF from **16** (Scheme 4). Amine **20** was prepared by reduction of **12a** with Pd/C catalyst at 30 °C, H_2_, 1 atm in an H-Cube Pro reactor (Scheme 4).

### 2.5 Antiparasitic assays

#### 2.5.1 Maintenance of *G. lamblia*, *E. histolytica* and *T. vaginalis*

Trophozoites of *G. lamblia* strains (metronidazole sensitive (MtzS) line WB and the MtzR line 713-M3 [20], [21], *E. histolytica* strain HM1:IMSS and *T. vaginalis* strain F1623 [15] were axenically maintained in TYI-S-33 medium supplemented with penicillin (100 U/mL) and streptomycin (100 μg/mL) [22]. All experiments were performed using trophozoites harvested during the logarithmic phase of growth.

#### 2.5.2 EC_50_ assays

Compounds were screened for antiparasitic activity using an ATP-bioluminescence based assay for cell growth and survival [23], [24]. Briefly, 2.5 μL of 5 mM stocks were diluted with 17.5 μL sterile water to yield 625 μM working concentration of compounds. Three-fold serial dilutions were prepared yielding a concentration range of 0.25–625 μM. From this dilution plate, 4 μL volumes were transferred into 96-well microtitre plates followed by addition of 96 μL of trophozoites (5000 parasites) to yield a final 8-point concentration range spanning 0.01–25 μM. Assay plates were incubated for 24–48 h at 37 °C in the GasPak™ EZ Anaerobe Gas Generating Pouch Systems (VWR, West Chester, PA) to maintain anaerobic condition throughout the incubation period. Viable cell numbers were determined in triplicate using the CellTiter-Glo Luminescent Cell Viability Assay [23].

### 2.6 MIC assays

*Clostridium difficile* strains (630, ATCC BAA-1382 and NAP1/027, ATCC BAA-1803) were maintained as previously described [16]. The minimum inhibition concentration (MIC) was determined according to the CLSI Methods with modifications in broth and inoculum for *C. difficile* [16], [25], [26]. Briefly, compounds and control antibiotics were serially diluted two-fold in 96-well plates (Non-binding surface, Corning). The plates were placed in a Coy anaerobic chamber (5% H_2_, 10% CO_2_, 85% N_2_) overnight to reduce the medium. *C. difficile* bacteria from BHIS(TA) agar plates were cultured anaerobically in BHIS at 37 °C overnight. A sample of culture was then diluted 40-fold in BHIS broth and incubated at 37 °C for approximately 4.5 h. The resultant mid-log phase culture (OD_600_ = 0.4–0.6) was diluted to a final concentration of ∼1 × 10^6^ CFU/mL, then 50 μL was added to each well of the compound-containing 96-well plates, yielding a final cell concentration of 5 × 10^5^ CFU/mL and final volume of 100 μL with 3% maximum DMSO concentration. Compound concentration ranged from 64 to 0.03 μg/mL. An antibiotic standard, a positive growth control (no compound) and a sterility control (no bacteria) were included on each 96 well plate. Plates were covered and incubated at 37 °C for 24 h. MICs for each strain were determined as the lowest concentration without visible growth. Variance between replicates was typically within one 2-fold dilution. Median MICs are reported with a range given when the median MIC was between two tested concentrations.

### 2.7 Cytotoxicity

Human HEK293 and HepG2 cells were seeded at 3000 and 5000 cells per well in 384-well plates, respectively. Cells were cultured in Dulbecco's modified Eagle's medium with 10% FBS for 24 h at 37 °C, 5% CO_2_. A dilution series of compounds was added, with the highest concentration of 100 μM. The final concentration of DMSO in culture media was 0.5%, which showed no effect on cell growth. After 24 h incubation with the compounds, 5 μM resazurin was added into each well and incubated at 37 °C for 2 h. As a negative control, 1% Triton X-100 was added into the culture media to lyse all of the cells. The fluorescence intensity was read using Polarstar Omega with excitation/emission 560/590 nm. Data were analysed with GraphPad Prism 6 software (La Jolla, California USA) to calculate CC_50_ values.

### 2.8 Correlation of compound properties with activity

A correlation matrix between compound activity and physicochemical properties was calculated using Excel correlation analysis (Supplementary Table 2). AlogP, logD, MW, logS and tPSA were calculated from the 2D structure of the compounds, using Pipeline Pilot (Accelrys, Version 9.1.0.13). Antimicrobial activity was expressed as -log_10_ values of MIC or EC_50_, using average MIC (mol L^−1^) of MtzS *C. difficile* ATCC BAA-1382 and ATCC BAA-1803 strains and EC_50_ (mol L^−1^) against *G. lamblia* WB strain, *E. histolytica* HM1:1MSS strain and *T. vaginalis* F1623 strain. Correlations of determination (R^2^) between compound activities and logD, MW or logS were determined by linear regression analysis in GraphPad Prism 6 software (La Jolla, California USA) (Supplementary Figs. 1–3).

### 2.9 Microscopy

The effect of compounds on *G. lamblia* WB growth and survival was examined by light microscopy. Briefly, stock compounds were diluted in DMSO (100%) to give 400 × final concentration of compound. An aliquot of 2.5 μL of working stock was added to each well of a 24 well tissue culture clear bottom plate (Corning, 3524), followed by trophozoites (1 mL, 50,000 parasites/mL) to yield a final concentration of 3 × EC_50_. Metronidazole (3 × EC_50_) served as a positive control. Media only wells were included as a sterility control, and vehicle only (0.25% DMSO) was included as a control for growth. Assay plates were incubated for 48 h at 37 °C in the GasPak™ EZ Anaerobe Gas Generating Pouch Systems (VWR, West Chester, PA) to maintain anaerobic conditions throughout the incubation period. The assays were performed in triplicate (3 wells/treatment). Growth inhibition was visualised by phase contrast microscopy (200 × magnification) (Carl Zeiss).

### 2.10 Plasma protein binding

Plasma Protein Binding (PPB) was performed using an Ultrafiltration method [27], [28]. Fresh frozen human plasma was pooled from 0 Positive (Product Number 2799882) and 0 Negative (Product number 5398256) blood from the R & D division of the Australian Red Cross Blood Services (Brisbane). Stock solutions (2.5 mM) of the test compounds were prepared in DMSO. Test compounds (5 μM) were incubated in 100% human plasma at 37 °C for 30 min (1 mL volume). For unfiltered samples, an aliquot (50 μL) was removed, diluted with PBS (50 μL) and quenched with ice-cold precipitating solution comprising 0.5 μM carbutamide MS internal standard in acetonitrile: methanol: formic acid (1: 1: 0.001). Samples were incubated at 4 °C for 30 min, then centrifuged at 14,000 × g for 8 min, with the clear supernatant transferred to a vial for LC/MS/MS analysis. For filtered samples, the plasma sample (250 μL) was filtered using Amicon Ultra-0.5 Centrifugal Filter Devices 30K NMWL at 14,000 × g for 7 min and then an aliquot (50 μL) was processed as described for unfiltered samples. The fraction of unbound compound was calculated by determining the concentration of the filtered sample and the concentration of unfiltered sample. All samples were tested in triplicate with sulfamethoxazole as a control. LC/MS/MS parameters are detailed in the supplementary information (Supplementary Table 1).

### 2.11 Metabolic stability

Metabolic stability studies were performed with human liver microsomes (HMMC-PL, Lot# PL050B-B, Thermo Fisher Scientific USA) with test compound (5 μM) degradation monitored by LC/MS/MS analysis. Stock solutions (2.5 mM) of the test compounds were prepared in DMSO. The reaction mixture containing test compound (5 μM) and liver microsomes (1 mg/mL) in 100 mM potassium phosphate buffer pH 7.4 was preincubated at 37 °C (1 mL volume). The reaction was initiated by addition of NADPH solution (cofactor) in potassium phosphate buffer (final concentration 1 mM). Aliquots (180 μL) from the reaction mixture were withdrawn (t = 0, 10, 30, 60 and 120 min) and quenched by adding ice-cold stop solution (540 μL) comprising 0.5 μM carbutamide internal standard in acetonitrile: methanol: formic acid (1: 1: 0.001). Reaction samples were incubated at 4 °C for 30 min, centrifuged at 14,000 × *g* for 8 min and the clear supernatant was transferred to a vial for LC/MS/MS analysis. The percentage of compound metabolised at different times was calculated as a percentage of the levels at the start of incubation (t = 0 min sample). Matrix blank was also prepared as a control. All samples were tested in triplicate except for the control samples (without NADPH), matrix blank and verapamil standard (time points = 0, 10 and 30 min). LC/MS/MS parameters are detailed in the supplementary information (Supplementary Table 1).

## 3 Results and discussion

### 3.1 Biological activity of 1-methyl-5-nitroimidazole carboxamides

The antiparasitic activity of the 1-methyl-5-nitroimidazole carboxamides **8a-k** against *G. lamblia* and *E. histolytica* was first assessed (Table 1). All of the 5-nitroimidazoles were active against MtzS *G. lamblia* WB, with EC_50_ values ranging from 1.6 μM to 4.9 μM (cf. metronidazole EC_50_ = 6.1 μM). Compounds **8f** (R = NHCH_2_(2-pyridinyl)) and **8h** (R = morpholine) were the most active derivatives (EC_50_ = 1.6 μM) with ∼4-fold greater potency than metronidazole, suggesting that polar groups conferred superior activity. The aromatic substituted benzyl amides (**8a-d**) and **8e** (R = NHCH_2_CH_2_(4-Me-Ph)) all had similar activity (EC_50_ = 2.8–3.5 μM), demonstrating that carboxamide groups with different aromatic substitution patterns are tolerated at the 2′-imidazole ring position. The aliphatic cyclic secondary amides of **8j** (R = NH-cyclopropyl) and **8k** (R = NH-cyclohexyl) were also both well tolerated, as were the tertiary amide groups of **8g** (R = NMe_2_) and **8i** (R = pyrrolidine). Encouragingly, a number of compounds were also active against MtzR *G. lamblia* 713M3 (Table 1). For example, **8c** (R = NHCH_2_(3-OCF_3_-Ph), **8d** (R = NHCHMe-(4-F-Ph)), **8f** (R = NCH_2_(2-pyridinyl)), **8i** (R = pyrrolidine) and **8k** (R = NH-cyclohexyl) were 3–12-fold more active than metronidazole against this MtzR *G. lamblia* strain (EC_50_ 1.5–5.1 μM, cf. metronidazole EC_50_ = 18 μM).

Carboxamides **8a-k** generally exhibited moderate activity against *E. histolytica* with a wide range of EC_50_ values from 3.7 μM to 22 μM (Table 1). Compound **8k** (R = NH-cyclohexyl) was the most potent (EC_50_ = 3.7 μM) with similar activity to metronidazole (EC_50_ = 5.0 μM), a notable difference in SAR compared to the preference for greater polarity against *G. lamblia*. However, the majority of the series, including the smaller lipophilic cyclopropyl amide **8j**, were 2–4-fold less potent than metronidazole, leaving room for further optimisation.

For comparison, we next tested the new derivatives against *T. vaginalis*, which was the original indication for nitroimidazole carboxamides [17]. Several of the compounds had potent activity against *T. vaginalis*, with EC_50_ values that were similar to metronidazole (EC_50_ = 0.6–1.4 μM cf. metronidazole EC_50_ = 0.8 μM). Notably, the smaller, more polar and nonaromatic side chains of **8g-j** were favourable, with activities from 0.6 to 1.7 μM, which was consistent with **8g-h** having activity in an *in vivo* mouse model of *T. vaginalis* infection [17]. Similarly, an ionisable aromatic group of compound **8f** (R = CH_2_-(2-pyridinyl)) was beneficial (EC_50_ = 1.4 μM). In contrast the non-polar, aromatic benzyl (**8a-d**) and phenethyl (**8e**) substituents resulted in comparatively weak activity against *T. vaginalis* (EC_50_ = 3.8–13 μM).

Since nitroimidazole carboxamides contain a 5-nitroimidazole warhead similar to metronidazole, a common treatment for infections caused by both parasites and anaerobic bacteria, we also determined the antimicrobial activity of **8a-k** against the anaerobic bacterium *C. difficile*. *C. difficile* is an anaerobic Gram-positive bacterium that infects the colon and causes inflammation and diarrhoea [29], similar to symptoms of *G. lamblia* and *E. histolytica* infection. Surprisingly, none of the 1-methyl-5-nitroimidazole carboxamides **8a-k** had significant activity against the 630 or NAP1/027 strains of *C. difficile* (MIC ≥ 32 μg/mL) whereas metronidazole was quite potent (MIC = 0.5 μg/mL) (Table 1, Supplementary Table 3). Therefore this compound series, containing the 1-methyl-5-nitroimidazole core, exhibited greater selectivity toward anaerobic protozoan parasites compared to the anaerobic bacteria *C. difficile*, suggesting differences between the parasitic and bacterial proposed mechanisms of activation of the nitroimidazole carboxamides, or possibly differences in cellular uptake.

The compound series **8a-k** was not cytotoxic against human kidney or liver cell lines at the highest concentration tested (CC_50_ > 100 μM), so the calculated minimal selectivity indices ranged from >16 to >63 (Table 1).

### 3.2 Biological activity of 4(5)-nitroimidazoles

The promising activity of the 1-methyl-5-nitrocarboxamide series as antiparasitic agents led us to explore the nitroimidazole carboxamide scaffold further to understand the SAR and determine whether the activity could be improved. Hoff [17] previously reported the influence of alkyl and hydroxyl alkyl groups at the 1′-imidazole nitrogen position on antiparasitic activity but did not describe the corresponding compound series in which the 1′-imidazole nitrogen lacks substitution. Hence we generated an analogous series of 4(5)-nitroimidazole carboxamides (**12a-k**) with the unsubstituted 1-position and matched carboxamide R groups to **8a-k**, and tested them for antiparasitic and antimicrobial activity. Compared to the 1-methyl-5-nitro series, the presence of an acidic imidazole proton in the 4(5)-nitro series permits ring tautomerism, which may alter the reduction potential of the nitro group. Furthermore, the small structural change will influence other physicochemical parameters (e.g. polar surface area and logS) which is likely to be reflected in different SAR profiles between the two series.

In general the 4(5)-nitroimidazole carboxamides **12a-k** exhibited improved activity against *G. lamblia* and *E. histolytica* relative to their 5-nitroimidazole counterparts **8a-k** (Table 1). For *G. lamblia*, the aromatic benzyl amides **12a-d**, phenethyl **12e** and cyclohexyl **12k** groups were very potent (EC_50_ = 0.1–0.6 μM). In contrast **12f** (R = NH—CH_2_(2-pyridinyl)), **12g** (R = NMe_2_) and **12h** (R = morpholine) were 3.5–5.5-fold less active than the respective 1-methyl analogues **8f**, **8g** and **8h**. Compounds **12i** (R = pyrrolidine) and **12j** (R = NH-cyclopropyl) maintained similar activity (EC_50_ = 3.4 and 5 μM, respectively) to the 1-methyl analogues **8i** and **8j**. A number of compounds with substituted benzyl groups (**12a-b** and **12d**) and the phenethyl derivative **12e** also displayed good activity against MtzR *G. lamblia* (EC_50_ ≤ 2.5 μM).

For *E. histolytica*, the 4(5)-nitromidazole carboxamides were overall more potent than the 1-methyl series, with activities ranging from 1.7 to 15 μM for compounds **12a-k** compared to 3.7–22 μM for the **8a-k** series. Several compounds in the **12a-k** series (**12d** (R = NHCHMe-(4-F-Ph)), **12e** (R = NHCH_2_CH_2_(4-Me-Ph)), **12g** (R = NMe_2_) and **12k** (R = NH-cyclohexyl)) were 2–3-fold more potent than metronidazole, while all of the other compounds had similar activity to metronidazole, except for the pyridine **12f** that was the least potent compound (EC_50_ = 15 μM).

In contrast to their improved activity against *G. lamblia* and *E. histolytica*, compounds **12a-k** were not overall more active than **8a-k** against *T. vaginalis* (Table 1). The SAR was relatively flat: the trend for improved potency with more polar substituents seen with series **8a-k** disappeared. The most potent compound was **12d** (R = NHCHMe(4-F-Ph)) with EC_50_ = 0.6 μM. The other aromatic benzyl compounds **12a-12c**, **12e** and the pyrrolidine **12i** had similar activity (EC_50_ = 1.2–2.3 μM), but were generally 2–3 fold less potent than **12d**. Interestingly, the absence of *N*-substitution on the imidazole ring for **12a-k** also greatly improved activity against both the 630 and NAP1/027 strains of *C. difficile* (MIC = 0.5–16 μg/mL), whereas the 1-methyl-5-nitro series were all essentially inactive (≥32 μg/mL) (Table 1, Supplementary Table 3). Small lipophilic and polar 2′-carboxamide substituents were preferred in the case of *C. difficile*. For example, **12j** (R = NH-cyclopropyl) was the most active derivative against *C. difficile* (MIC = 1 μg/mL), although less active than metronidazole (MIC = 0.5 μg/mL), while **12f-i** (pyridine, dimethyl, morpholine and pyrrolidine derivatives) had MIC = 2 μg/mL. In contrast, the aromatic benzyl **12a-d**, phenethyl **12e** and cyclohexyl **12k** compounds were less active (MIC = 4–16 μg/mL). To further understand this preference for activity against *C. difficile*, additional small, polar amides **12l-o** were synthesised. These included **12l** (R = NH_2_), **12m** (R = NHMe) and two compounds inspired from the side chain of metronidazole: **12n** (R = NHCH_2_CH_2_OH) and **12o** (R = NMeCH_2_CH_2_OH). Compounds **12l-m** and **12o** gave results that supported the previous trend observed against *C. difficile* (MIC = 0.5–2 μg/mL), while **12n** (R = NHCH_2_CH_2_OH) was less active (MIC = 8–16 μg/mL). These additional compounds **12l-o** had weak to no activity against the parasites.

The majority of the 4(5)-imidazole series **12a-o** were not cytotoxic at the highest concentration tested (CC_50_ > 100 μM) against mammalian liver or kidney cell lines. The only compound found to show cytotoxicity was **12b** (R = NHCH_2_(4-OCF_3_-Ph)) against the HepG2 liver cell line (CC_50_ = 93 μM), but the selectivity index (SI = 465) relative to *G. lamblia* activity remained excellent.

#### 3.2.1 Phenotypic effect of 4(5)-nitroimidazole 12a on G. lamblia

**Fig. 2:**
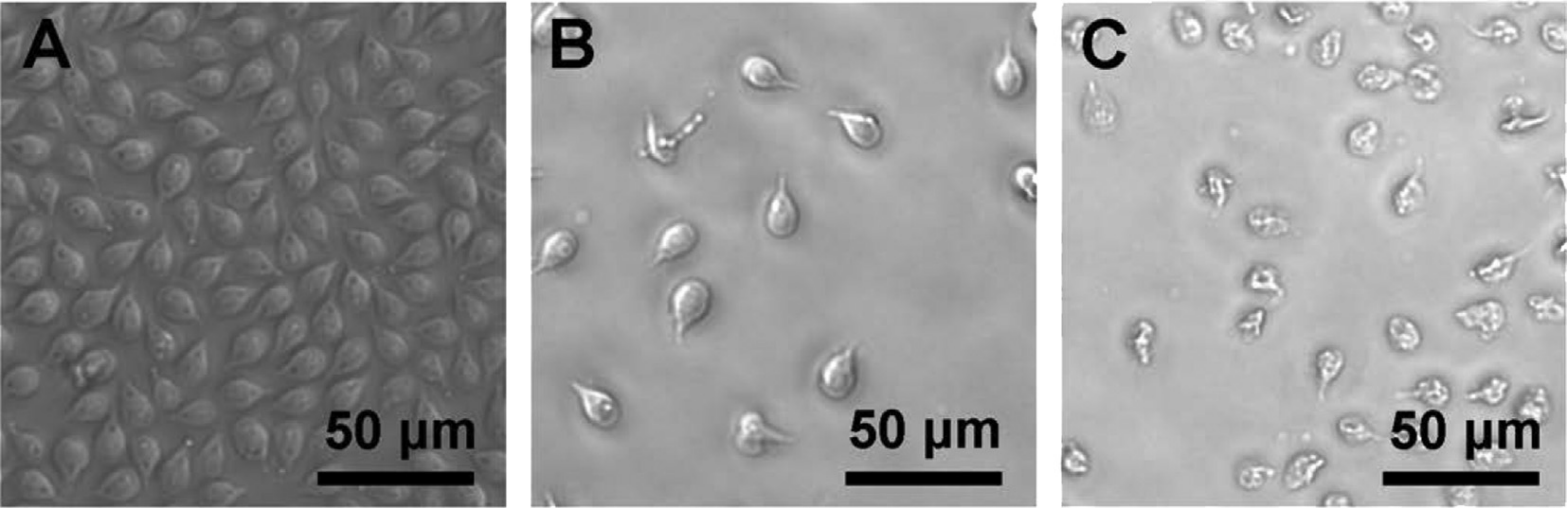
Compound **12a** inhibited *Giardia lamblia* trophozoites growth A) vehicle (DMSO), B) metronidazole (3 × EC_50_ = 18 μM) and C) **12a** (3 × EC_50_ = 1.5 μM).

Microscopy was used to visually examine the impact of one of the most potent compounds, the 4(5)-nitroimidazole **12a** (R = NHCH_2_(4-F-Ph)), on *G. lamblia* trophozoites. Parasite cell growth was similarly inhibited by treatment with 3 × EC_50_ of either metronidazole (18 μM) or compound **12a** (1.5 μM) relative to the vehicle control (which produced a confluent cell layer under the test conditions). The morphology of *G. lamblia* treated with **12a** was altered, while the morphology of the metronidazole-treated cells remained similar to the vehicle control (Fig. 2). A prior study by Tejman-Yarden et al. reported that metronidazole slowed the rate of oscillation of the *Giardia* flagella, while auranofin, a compound with a proposed different mode of action, caused cell blebbing [24]. The different morphology of the *G. lamblia* treated with the 4(5)-nitroimidazole carboxamide **12a** may indicate an additional mode of action compared to metronidazole.

#### 3.2.2 Influence of physicochemical properties on compound activity in the 4(5)-nitromidazole compound series

We observed improved activity profiles of 4(5)-nitroimidazoles relative to the corresponding analogue in the 5-nitroimidazole series against *G. lamblia*, *E. histolytica* and *C. difficile*, but not *T. vaginalis*. In addition, the 4(5)-nitroimidazoles with the most potent activity against *G. lamblia* differed significantly from the compounds with the most potent activity against *C. difficile*. To better understand the relationship between biological activity and physicochemical properties, the correlation coefficients (r) were determined between a range of calculated compound properties (AlogP, logD, molecular weight, logS and topological polar surface area) and biological activity against the different organisms (Supplementary Table 2). *G. lamblia* activity was positively correlated with AlogP (r = 0.94), logD (r = 0.93) and MW (r = 0.82). A negative correlation with LogS (r = −0.92) was also observed, while there was no meaningful relationship with tPSA (r = 0.06). Nearly identical results were obtained with logP and logD values as only **12f** (R = NHCH_2_(2-pyridinyl)) contained an ionisable group. Moderate to weak correlations were observed between *E. histolytica* or *T. vaginalis* activity and compound properties (AlogP, logD, MW, logS and tPSA). In contrast, *C. difficile* activity was positively correlated with LogS (r = 0.72), negatively correlated with AlogP (r = −0.72), logD (r = −0.72) and MW (r = −0.75) and poorly correlated with tPSA (r = −0.23), supporting the qualitative observations made from examination of the SAR.

**Fig. 3:**
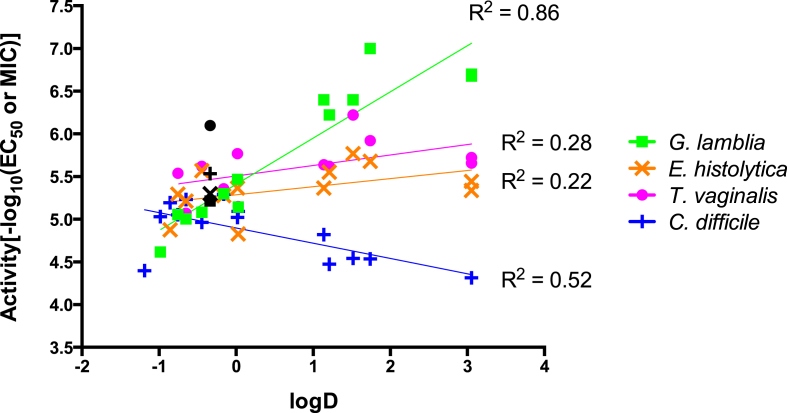
Activity vs logD of active 4(5)-nitroimidazoles. Compounds were classified as active against *G. lamblia* and *E. histolytica* with EC_50_ < 50 μM and were considered active against *T. vaginalis* with EC_50_ < 20 μM. All 4(5)-nitroimidazoles were classified as active against *C. difficile* (MIC ≤ 16 μg/mL). Metronidazole (black symbols) is shown for comparison.

To quantify the extent that the variability in activity against each organism was dependent on logD, MW and logS, the coefficient of determination (R^2^) was next calculated (Fig. 3, Supplementary Figs. 1–3). This analysis supported the correlation between *G. lamblia* activity and logD, MW and logS properties of the compounds (R^2^ ranged from 0.67 to 0.86) (Fig. 3, Supplementary Figs. 1–3). No correlation was found for *E. histolytica* and *T. vaginalis* activity and compound properties (R^2^ ranged from 0.15 to 0.28) (Fig. 3, Supplementary Figs. 1–3). In contrast, a weak correlation between *C. difficile* activity and logD, MW and logS was observed (R^2^ ranged from 0.47 to 0.56) (Fig. 3, Supplementary Figs. 1–3), demonstrating greater variability in the data that was not accounted for by changes to logD, MW or logS.

To summarise, while activity against *G. lamblia* was improved by increasing logD, MW and decreasing logS, this trend was not apparent for *E. histolytica* or *T. vaginalis*. In contrast, activity against *C. difficile* was weakly improved with compounds with lower logD, MW and greater logS.

### 3.3 Biological activity of 4-nitroimidazoles

Given the potent activity of the 4(5)-nitroimidazoles relative to the 1-methyl-5-nitroimidazoles, we were interested to determine the activity of 4-nitroimidazole carboxamide analogues, since research by Trunz et al. showed that 4-nitroimidazoles can have potent antiparasitic activity [30]. We therefore prepared a series of 4-nitroimidazole carboxamides **13a-g**. Since polar substituents were favourable for activity against *G. lamblia* in the 5-nitroimidazole series (though not the 4(5)-nitroimidazole series), compounds were synthesised with the 2′-substituent as a primary carboxamide group with the 1′-ring position substituted with benzyl, phenethyl, heteroaromatic pyridine, cyclopropyl and cyclohexyl groups. The compounds were found to have selective activity against *G. lamblia* (Table 2). Several of these compounds, including **13b** (R = CH_2_(4-OCF_3_-Ph)), **13c** (R = CH_2_(3-OCF_3_-Ph)), and **13g** (R = CH_2_(cyclohexyl)) displayed activities similar to metronidazole (EC_50_ = 4.1–8.4 μM cf. metronidazole EC_50_ = 6.1 μM), while being non-cytotoxic to human liver or kidney cell lines (CC_50_ > 100 μM). Interestingly, none of the **13a-f** series had activity against *E. histolytica* or *C. difficile*.

To determine the relative influence of the 2′ position on the potency and selectivity for *G. lamblia*, we next modified the 2′ position to methyl amide **14a**, dimethyl amide **14b**, ethyl ester **14c**, hydroxamide **14d** and hydrazide **14e** while maintaining the 1′ ring position with the preferred CH_2_(4-OCF_3_-Ph) group. Compounds **14a** (R = NHMe) and **14b** (R = NMe_2_) were the most active against *G. lamblia* (EC_50_ = 3.4 and 2.7 μM, respectively), slightly more potent than the primary amide **13b** (EC_50_ = 4.1 μM) and metronidazole (EC_50_ = 6.1 μM). Compounds **14c** (R = COOEt), **14d** (R = NHOH) and **14e** (R = NHNH_2_) had similar or slightly reduced activity relative to **13b** (R = NH_2_). Therefore different 2′ substituents were tolerated for activity against *G. lamblia*. Although compound series **14** displayed improved activity compared to compound series **13** against *E. histolytica* (EC_50_ = 10–45 μM vs >50 μM) and *C. difficile* (MIC = 16 to ≥64 μg/mL vs >64 μg/mL), the overall activity profile of both series remained considerably inferior to metronidazole (Table 2, Supplementary Table 4) and compounds within series **8** and **12**. These results demonstrate that *G. lamblia* is selectively sensitive to 4-nitromidazoles, suggesting differences in the nitro-reduction activation and/or uptake of 4-nitroimidazoles compared to *E. histolytica* and *C. difficile*.

### 3.4 Desnitro and amine derivatives

5-Nitroimidazole antimicrobial agents are pro-drugs that are activated by reduction of the nitro group to reactive intermediates that cause cellular damage [31]. The reduction step is catalysed by organism specific oxidoreductase enzymes, confounding target based drug design and enzymatic assays as approaches to drug development. In *G. lamblia*, the enzymes pyruvate ferredoxin oxidoreductase, nitroreductase 1 and thioredoxin reductase 1 have been implicated in the reductive activation of metronidazole [32]. Since the nitro group is key to the mode of action of metronidazole, we sought to establish whether this functional group is also important for the activity of these nitroimidazole carboxamides, which are thought to act by similar mechanisms as metronidazole. Thus, we prepared desnitro analogues **17** and **18** and the reduced amine derivative **20** (Scheme 4). As hypothesised, all three compounds displayed no discernable activity against parasites or *C. difficile*, supporting the importance of the nitro group in the mode of action of nitroimidazole carboxamides (Supplementary Table 5).

### 3.5 Plasma protein binding and microsome stability

Metronidazole is essentially 100% orally absorbed [33], yet exposure of *G. lamblia* parasites to the drug in the intestinal tract after the initial absorption period continues to occur by biliary excretion and enterohepatic circulation [34]. Oral absorption is also necessary for treatment of invasive amebiasis, underlying the importance of adequate absorption of nitro drugs for *in vivo* efficacy. To delineate preliminary ADME characteristics of the new nitroimidazole carboxamide compounds, we determined their plasma protein binding and microsome stability, as these properties are likely to influence compound half-life and free drug available at the sites of infection.

Binding to human plasma proteins was measured for several 4(5)- and 5-nitroimidazole carboxamide matched pairs, including **8a** and **12a** (R = NHCH_2_(4-F-Ph)), **8k** and **12k** ((R = NH-cyclohexyl)) and **8h** and **12h** (R = morpholine) (Table 3). Plasma protein binding varied depending on the 1′- and 2′-substituents. The 4-F-benzylamide (**8a** and **12a**) and cyclohexylamide (**8k** and **12k**) imidazoles were highly bound to plasma proteins (≥94%) regardless of the 1′-substituent (H or Me), with the plasma protein binding for 1′-H derivatives slightly greater in each instance. In contrast, the morpholine group of **8h** ameliorated plasma protein binding (9% bound) for the 5-nitroimidazole but the 4(5)- matched pair **12h** displayed high plasma protein binding, while metronidazole was almost completely unbound (<5% bound). The contrast in plasma protein binding between **8h** and **12h** could be explained by the acidic nature of the imidazole N—H bond observed in the proton NMR (N*H* δ ∼14.3 ppm) and tendency for plasma proteins such as human serum albumin to bind anionic compounds [27]. While both metronidazole and tinidazole are mostly unbound to plasma proteins [35], tizoxanide, the active metabolite of the prodrug nitazoxanide, is highly plasma protein bound [36]. The influence of plasma protein binding on the free drug concentration at the site of infection is also related to other complex factors, including metabolism, distribution and half-life, and further *in vivo* efficacy experiments are necessary to determine the impact of high plasma protein binding on *in vivo* efficacy in this series [37].

The metabolic stability of a compound influences the concentration of drug available in the circulation for treatment of invasive amebiasis, and for prolonged exposure of *G. lamblia* to drug treatment via enterohepatic recirculation pathways. Therefore, we measured the human liver microsome stability for the 4(5)- and 5-nitroimidazoles matched pairs **8a** and **12a** (R = NHCH_2_(4-F-Ph)) and **8h** and **12h** (R = morpholine) as these compounds showed good potency and a range of plasma protein binding. All of the compounds were metabolically stable after 2 h incubation with human liver microsomes (Table 3). This result was comparable to metronidazole, suggesting that the compounds have favourable metabolic stability and that different 2′ substituents were tolerated.

## 4 Conclusion

New nitroimidazole carboxamides were identified with activity against the pathogenic parasites *G. lamblia*, including a metronidazole-resistant strain, and *E. histolytica*. The most potent derivatives displayed a wide range of plasma protein binding and were metabolically stable, with comparable stability to metronidazole. The rediscovery and derivatisation approach taken in this study could be applied to other ‘forgotten’ compounds to facilitate rapid research and development of new antiparasitic agents.

## Figures and Tables

**Table 1:** Activity of 1-methyl-5-nitroimidazole carboxamides and 4(5)-nitroimidazole carboxamides against *G. lamblia*, *E. histolytica*, *T. vaginalis* and *C. difficile*.

			EC_50_ (μM) (pEC_50_ ± SE)				MIC (μg/mL)	CC_50_ (μM) (pCC_50_ ± SE)		S.I^a^ (CC_50_/EC_50_)
*G. lamblia*		*E. histolytica*	*T. vaginalis*	*C. difficile*^b^	HEK 293	Hep G2
WB	713M3	HM1:IMSS	F1623	630
No.	X	R	MtzS	MtzR	MtzS	MtzS	MtzS
**1**	Metronidazole		6.1 (5.21 ± 0.05)	18 (4.74 ± 0.02)	5.0 (5.30 ± 0.03)	0.8 (6.1 ± 0.07)	0.5	>100	>100	>16
**8a**	Me	NHCH_2_(4-F-Ph)	3.5 (5.46 ± 0.03)	13 (4.89 ± 0.11)	9.7 (5.01 ± 0.01)	5.2 (5.28 ± 0.11)	>64	>100	>100	>29
**8b**	Me	NHCH_2_(4-OCF_3_-Ph)	3.0 (5.52 ± 0.03)	8.8 (5.06 ± 0.29)	14 (4.85 ± 0.02)	13 (4.88 ± 0.01)	32	>100	>100	>33
**8c**	Me	NHCH_2_(3-OCF_3_-Ph)	3.1 (5.51 ± 0.02)	1.5 (5.84 ± 0.69)	17 (4.77 ± 0.02)	8.1 (5.09 ± 0.09)	32–64	>100	>100	>32
**8d**	Me	NHCHMe(4-F-Ph)	2.9 (5.54 ± 0.03)	1.9 (5.73 ± 0.39)	10 (5.00 ± 0.03)	3.8 (5.42 ± 0.24)	64	>100	>100	>34
**8e**	Me	NHCH_2_CH_2_(4-Me-Ph)	2.8 (5.55 ± 0.03)	>20 (<4.70)	13 (4.89 ± 0.03)	8.1 (5.09 ± 0.16)	>64	>100	>100	>36
**8f**	Me	NHCH_2_(2-pyridinyl)	1.6 (5.80 ± 0.03)	4.1 (5.39 ± 0.29)	14 (4.85 ± 0.14)	1.4 (5.84 ± 0.09)	64	>100	>100	>63
**8g**	Me	N(Me)_2_	2.4 (5.62 ± 0.03)	>20 (<4.70)	14 (4.85 ± 0.03)	1.3 (5.89 ± 0.30)	32	>100	>100	>42
**8h**	Me	morpholine	1.6 (5.80 ± 0.03)	11 (4.95 ± 0.20)	14 (4.85 ± 0.02)	0.6 (6.24 ± 0.34)	32	>100	>100	>63
**8i**	Me	pyrrolidine	2.9 (5.54 ± 0.04)	4.0 (5.40 ± 0.33)	22 (4.66 ± 0.02)	1.7 (5.76 ± 0.17)	64	>100	>100	>34
**8j**	Me	NH-cyclopropyl	3.4 (5.47 ± 0.04)	8.6 (5.06 ± 0.23)	12 (4.92 ± 0.03)	1.1 (5.96 ± 0.30)	>64	>100	>100	>29
**8k**	Me	NH-cyclohexyl	4.9 (5.31 ± 0.04)	5.1 (5.30 ± 0.20)	3.7 (5.43 ± 0.03)	11 (4.97 ± 0.14)	>64	>100	>100	>20

**12a**	H	NHCH_2_(4-F-Ph)	0.5 (6.28 ± 0.10)	2.4 (5.63 ± 0.35)	3.6 (5.44 ± 0.06)	2.3 (5.63 ± 0.17)	4	>100	>100	>250
**12b**	H	NHCH_2_(4-OCF_3_-Ph)	0.2 (6.61 ± 0.03)	2.5 (5.60 ± 0.38)	4.5 (5.35 ± 0.06)	2.2 (5.65 ± 0.23)	16	>100	93 (4.03 ± 0.07)	>500/465
**12c**	H	NHCH_2_(3-OCF_3_-Ph)	0.2 (6.70 ± 0.03)	15 (4.83 ± 0.11)	3.6 (5.44 ± 0.01)	1.9 (5.72 ± 0.17)	16	>100	>100	>500
**12d**	H	NHCHMe(4-F-Ph)	0.4 (6.40 ± 0.6)	1.3 (5.90 ± 0.31)	1.7 (5.77 ± 0.04)	0.6 (6.23 ± 0.14)	8	>100	>100	>250
**12e**	H	NHCH_2_CH_2_(4-Me-Ph)	0.1 (7.00 ± 0.04)	1.4 (5.87 ± 0.39)	2.1 (5.68 ± 0.04)	1.2 (5.92 ± 0.16)	8	>100	>100	>1000
**12f**	H	NHCH_2_(2-pyridinyl)	7.2 (5.14 ± 0.03)	>20 (<4.70)	15 (4.82 ± 0.02)	7.1 (5.15 ± 0.11)	2	>100	>100	>14
**12g**	H	N(Me)_2_	8.3 (5.08 ± 0.02)	>20 (<4.70)	2.7 (5.57 ± 0.03)	2.4 (5.63 ± 0.11)	2	>100	>100	>12
**12h**	H	morpholine	8.8 (5.06 ± 0.02)	>20 (<4.70)	5.1 (5.29 ± 0.02)	2.9 (5.54 ± 0.05)	2	>100	>100	>11
**12i**	H	pyrrolidine	3.4 (5.47 ± 0.02)	13 (4.88 ± 0.11)	4.3 (5.37 ± 0.02)	1.7 (5.77 ± 0.11)	2	>100	>100	>29
**12j**	H	NH-cyclopropyl	5.0 (5.30 ± 0.03)	>20 (<4.70)	5.3 (5.28 ± 0.02)	4.4 (5.36 ± 0.05)	1	>100	>100	>20
**12k**	H	NH-cyclohexyl	0.6 (6.22 ± 0.03)	5.5 (5.26 ± 0.26)	2.8 (5.55 ± 0.04)	2.4 (5.61 ± 0.10)	8	>100	>100	>167
**12l**	H	NH_2_	>50 (<4.3)	>20 (<4.70)	13 (4.88 ± 0.03)	>20 (<4.70)	1	>100	>100	N/A
**12m**	H	NHMe	9.9 (5.00 ± 0.02)	>20 (<4.70)	6.1 (5.21 ± 0.02)	8.5 (5.07 ± 0.15)	0.5–1	>100	>100	>10
**12n**	H	NHCH_2_CH_2_OH	>50 (<4.3)	>20 (<4.70)	>50 (<4.3)	>20 (<4.70)	8	>100	>100	N/A
**12o**	H	NMeCH_2_CH_2_OH	24 (4.62 ± 0.05)	>20 (<4.70)	>50 (<4.3)	>20 (<4.70)	2	>100	>100	>4

a Selectivity Index: average cytotoxicity against HEK293 and HepG2 cell lines/*G. lamblia* WB activity(CC_50_/EC_50_).

b Similar results were obtained for *C. difficile* NAP/027 strain (Supplementary Table 3). EC_50_ minimum n = 3 EC_50_, pEC_50_ ± SE, MIC median of n = 4, CC_50_ n = 3, pCC_50_ ± SE.

**Table 2:** *In vitro* activity of 1-substituted 4-nitroimidazoles against *G. lamblia* and *E. histolytica*.

			EC_50_ (μM) (pEC_50_ ± SE)		MIC (μg/mL)	CC_50_ (μM) (pCC_50_ ± SE)		S.I^a^ (CC_50_/EC_50_)
*G. lamblia*	*E. histolytica*	*C. difficile*	HEK293	HepG2
No.	X	R	WB	HM1:IMSS	630
**1**	Metronidazole		6.1 (5.21 ± 0.05)	5.0 (5.30 ± 0.03)	0.5	>100	>100	>16
**13a**	CH_2_(4-F-Ph)	-NH_2_	>50 (<4.3)	>50 (<4.3)	>64	>100	>100	N/A
**13b**	CH_2_(4-OCF_3_-Ph)	-NH_2_	4.1 (5.39 ± 0.03)	>25 (<4.6)	>64	>100	>100	>22
**13c**	CH_2_(3-OCF_3_-Ph)	-NH_2_	8.4 (5.08 ± 0.05)	>50 (<4.3)	>64	>100	>100	>12
**13d**	CH_2_CH_2_(4-Me-Ph)	-NH_2_	>50 (<4.3)	>50 (<4.3)	>64	>100	>100	N/A
**13e**	CH_2_(2-pyridinyl)	-NH_2_	27 (4.57 ± 0.07)	>50 (<4.3)	>64	>100	>100	>3.7
**13f**	CH_2_-cyclopropyl	-NH_2_	>50 (<4.3)	>50 (<4.3)	>64	>100	>100	N/A
**13g**	CH_2_-cyclohexyl	-NH_2_	5.0 (5.30 ± 0.06)	>50 (<4.3)	>64	>100	>100	>20

**14a**	CH_2_(4-OCF_3_-Ph)	-NHMe	3.4 (5.47 ± 0.04)	30 (4.52 ± 0.03)	64–>64	>100	>100	>29
**14b**	CH_2_(4-OCF_3_-Ph)	-NMe_2_	2.7 (5.57 ± 0.04)	19 (4.72 ± 0.04)	64	>100	>100	>37
**14c**	CH_2_(4-OCF_3_-Ph)	-OEt	6.0 (5.22 ± 0.05)	51 (4.29 ± 0.03)	>64	>100	>100	>17
**14d**	CH_2_(4-OCF_3_-Ph)	-NHOH	5.1 (5.29 ± 0.04)	10 (5.00 ± 0.03)	16	36 (4.44 ± 0.06)	>100	7/>20
**14e**	CH_2_(4-OCF_3_-Ph)	-NHNH_2_	7.5 (5.12 ± 0.05)	45 (4.35 ± 0.03)	>64	>100	>100	>13

a Selectivity Index: average cytotoxicity of HEK293 and HepG2 cell lines/*G. lamblia* WB activity (CC_50_/EC_50_). EC_50_ minimum n = 3 EC_50_ (pEC_50_ ± SE), MIC median of n = 4, CC_50_ n = 3 (pCC_50_ ± SE).

**Table 3:** Human plasma protein binding and microsome stability for selected compounds.

No.	X	R	*G. lamblia* EC_50_ (μM)	Plasma protein binding (% bound)	Microsome stability (% remaining at 2 h)
**1**	Metronidazole		6.1	<5	100
**8a**	Me	NHCH_2_(4-F-Ph)	3.5	95	88
**12a**	H	NHCH_2_(4-F-Ph)	0.5	>99	94
**8h**	Me	morpholine	1.6	9	97
**12h**	H	morpholine	8.8	78	100
**8k**	Me	NH-cyclohexyl	4.9	94	N.D
**12k**	H	NH-cyclohexyl	0.6	99	N.D

Plasma protein binding sulfamethoxazole control = 68% bound (consistent with literature 66% bound [38]); microsome stability verapamil control = 25% remaining at 2 h (consistent with literature [39]).
