# Supplementary material for: Nitroimidazole carboxamides as antiparasitic agents targeting Giardia lamblia, Entamoeba histolytica and Trichomonas vaginalis
Source: Eur J Med Chem. 2016 Sep 14;120:353–62. doi: 10.1016/j.ejmech.2016.04.064 (PMC4920673; doi:10.1016/j.ejmech.2016.04.064)
Supplement: Supplementary file 1 [file mmc1.docx]

Supplementary Information

# Nitroimidazole carboxamides as antiparasitic agents targeting *Giardia lamblia*, *Entamoeba histolytica* and *Trichomonas vaginalis*

A. M. Jarrad, A. Debnath, Y. Miyamoto, K. A. Hansford, R. Pelingon, M. S. Butler, T. Bains, T. Karoli, M. A. T. Blaskovich, L. Eckmann, M. A. Cooper

Table of Contents

1 General Methods 2

2 Synthesis 3

2.1 General procedure A for amide coupling via acid chloride; **8a-k** 3

2.2 General procedure B for amide coupling with PyBOP coupling reagent; **12a-b**, **12g-I** and **17** 3

2.3 General procedure C for amide coupling via acid chloride; **12c-f**, **12j-k** 3

2.4 General procedure C for alkylation of imidazole; **13a-g**, **14a-c** 4

3 Spectra 24

4 Supplementary Methods 115

4.1 LC/MS/MS detection and analysis parameters for plasma protein binding and metabolic stability 115

5 Supplementary Results 116

5.1 Correlation of compound activity and properties 116

5.2 Activity of nitroimidazole carboxamides against *C. difficile* 119

5.3 Desnitro and amine derivatives were inactive against *G. lamblia*, *E. histolytica*, *T. vaginalis* and *C. difficile*. 120

6 References 121

## General Methods

Reagents and anhydrous solvents (dimethylformamide, dichloromethane, and acetonitrile) were used as received. Reactions requiring anhydrous conditions were performed under an inert atmosphere of nitrogen. Reactions were monitored by thin layer chromatography (TLC) or LCMS. Analytical TLC was performed on Merck TLC aluminium sheets pre-coated with Silica Gel 60 F-254 and compounds were visualised using UV lamp and potassium permanganate stain. Melting points were determined using a Gallenkamp melting point apparatus and are corrected to a standard curve of the measured and literature melting points of vanillin, acetyl salicylic acid, 3-phenoxybenzoic acid and caffeine standards. Analytical LCMS was performed on a Shimadzu LCMS using 0.05% formic acid in water as solvent A and 0.05% formic acid in acetonitrile as solvent B. LCMS conditions (solvent A = H_2_O + 0.05% formic acid, solvent B = acetonitrile + 0.05% formic acid): Standard (unspecified): Column Zorbax Eclipse XDB-Phenyl, 3.0×100mm, 3.5 μ: Flow: 1 mL/min: Gradient timetable: 0.00 min, 5% B; 3.00 min, 100% B; 3.7 min, 100% B; 5.00 min, 5% B. Waters Atlantis: Column: Waters Atlantis T3, 3.0 × 100mm, 3 μ: Flow 0.75 mL/min, Gradient Method 1: 0.00 min, 5% B; 3.30 min, 25% B; 3.50 min, 100% B; 4.00 min 100% B; 4.10 0% B; 5.00 min, 0% B, Gradient Method 2: timetable: 0.00 min, 0% B; 3.30 min, 5% B; 3.50 min, 100% B; 3.75 min 100% B; 3.80 min 0% B; 5.00 min, 0% B. Detection: UV at 254 nm, ELSD and electrospray MS. Compounds were purified by MPLC (Biotage Isolera or Grace Reveleris X2 chromatography systems) or by HPLC (Agilent Preparative HPLC 1260 Infinity Series). Commercially available cartridges were used for MPLC chromatography (Biotage SNAP cartridge HP-Silica 10 g, 25 g or 50 g, Reveleris 4 g or 12 g Silica (40 µm) cartridge or Reveleris C18 Reversed-Phase 12 g cartridge). Column for HPLC: Agilent XDB Phenyl 5 uM, 30 x 100 mm. All final products were obtained in >95% purity as determined by HPLC using UV at 254 nm, ESIMS and ELSD detection. NMR data were collected and calibrated in DMSO-*d*_6_ or CDCl_3_ with 0.05% TMS at 298K on a Varian Unity 400 MHz or Bruker Avance-600 MHz spectrometer. Where appropriate, ^1^H-coupling constants were examined using resolution enhancement with MestReNova software. Data are presented as follows: chemical shift (ppm), multiplicity (s = singlet, d = doublet, t= triplet, q = quartet, p = pentet, m = multiplet, br = broad), coupling constant (Hz) and integration. High resolution mass spectrometry (HRMS) was performed on a Bruker MicroTOF mass spectrometer using (+)-ESI calibrated to HCOONa. For compounds purified by reverse phase chromatography, the exact concentration of the compounds was determined by the quantitative NMR integration ‘PULCON’ experiment [1]. These settings were used for all PULCON experiments: relaxation delay of 30 s, 8 scans, 2 dummy scans, 90° pulse and temperature at 298 K. The formula weight obtained from PULCON experiments was used to calculate the percentage yields.

## Synthesis

### **General procedure A for amide coupling via acid chloride; 8a-k**

**

Oxalyl chloride (275 µL 3.25 mmol) was added to a stirred suspension of potassium carboxylate **6** (0.5 g, 2.12 mmol (estimated FW)) in anh. DCM (10 mL) at 0 °C under an atmosphere of N_2_. Anh. DMF (1 drop) was then added and the reaction was stirred at 0 °C to rt for 1.5 hr. Volatiles were then removed *in vacuo* and the residual oxalyl chloride was removed by co-evaporation with toluene to obtain acid chloride intermediate **7** as a brown solid. The solid was immediately suspended in anh. DCM (4.12 mL, 10 vol) and, in parallel, an aliquot (1 eq) was added to each respective stirred solution of amine (1.2 eq amine, 2 eq. TEA in 10 vol. DCM) at 0 °C under an atmosphere of N_2_. General work up procedure: the reaction was poured into water (100 vol), acidified to pH 1–4 with 2 M aq HCl and then extracted with DCM (100 vol). A portion of brine (25 vol) was added to aid layer separation. The aq layer was further extracted with DCM (2 x 100 vol). The combined organic layers were washed with brine, dried over anh. MgSO_4_ and filtered. Volatiles were then removed *in vacuo* to obtain crude residue.

### **General procedure B for amide coupling with PyBOP coupling reagent; 12a-b, 12g-I and 17**

To a stirred suspension of 5-nitro-1H-imidazole-2-carboxylic acid **10** (400 mg, 2.55 mmol), amine (1.5 eq) and PyBOP (1.15 eq) in DMF (40 vol) under N_2_ was added dropwise DIPEA (4 eq) causing the suspension to clear. After 20 min at rt, the reaction was poured into water (100 vol) and extracted with DCM (3 x 125 vol). The combined organic layers were washed with brine (300 vol), dried over anh. MgSO_4_, filtered and volatiles were removed in vacuo.

### General procedure C for amide coupling via acid chloride; 12c-f, 12j-k

******

To a stirred suspension of 5-nitro-1H-imidazole-2-carboxylic acid (1.87 g, 11.9 mmol) in anh. DCM (37 mL) cooled to 0 °C under N_2_ was added oxalyl chloride (2.0 mL, 23.8 mmol) followed by addition of DMF (2 drops). The reaction was allowed to warm to rt and stir overnight. A further portion of oxalyl chloride (500 µL, 5.95 mmol) and DMF (2 drops) was added and the reaction stirred for 1 hr. Volatiles were then removed *in vacuo* and the residual oxalyl chloride was removed by co-evaporation with toluene to afford the acid chloride as a colourless solid. The solid was immediately suspended in anh. DCM (31.3 mL), chilled to 0 °C under N_2_ and TEA (3.3 mL, 23.6 mmol) added. The clear blue-purple solution (1 eq. acid chloride, 2 eq. TEA, 15 vol DCM) was then added in parallel to respective stirred solutions of amine (1.2 eq) in DCM (5 vol.) cooled to 0 °C under N_2_. When the reaction was complete (generally within 15 mins), the reaction was poured into water (100 vol), acidified to pH 1-4 with 2 M aq HCl and then extracted with EtOAc (100 vol). A portion of brine (25 vol) was added to aid layer separation. The aqueous layer was further extracted with EtOAc (2 x 100 vol). The combined organic layers were washed with brine, dried over anh. MgSO_4_ and filtered. Volatiles were then removed *in vacuo* to afford the crude material.

### General procedure C for alkylation of imidazole; 13a-g, 14a-c

******

To a stirred solution of imidazole (1 eq) in anh. DMF (15 vol) was added K_2_CO_3_ (3 eq) followed by alkyl or benzyl halide (1.2 – 1.5 eq). Reactions were either stirred at rt, heated conventionally or heated in the microwave (80 – 120 °C) until LCMS revealed consumption of the imidazole stating material. Additional portions of alkylating agent were added as necessary to drive the reaction to completion. Isolation by precipitation: the reaction was poured into water (200 vol) and the precipitate was filtered. The solid was washed with water and pet. spirits and dried under vacuum to obtain the crude product. Aqueous work up: the reaction was poured into water (200 vol) and isolated by extraction with EtOAc (3 x 100 vol). The combined organic layers were washed with brine, dried with anh. MgSO_4_ and filtered. Volatiles were removed *in vacuo* to obtain the crude product.

Potassium 1-methyl-5-nitro-1H-imidazole-2-carboxylate; **6**

**

Potassium permanganate (650 mg, 4.14 mmol) was added portionwise to a stirred suspension of 1-methyl-5-nitro-1H-imidazole-2-methanol (503 mg, 3.18 mmol) in acetone (7.5 mL) cooled to -5 °C [2]. After the addition was complete, the reaction was warmed to rt and stirred for 4 hr. An addition portion of potassium permanganate (250 mg, 2.07 mmol) was then added with cooling to -5 °C, and the reaction stirred for an additional 1 hr at rt. The thick suspension was filtered and the solid was washed with water. The eluent was collected and volatiles were removed in vacuo before lyophilisation to obtain crude brown solid compound (0.73 g (77% w/w), 85%). The crude solid was used without further purification. LCMS (Waters Atlantis: Gradient Method 1): R_t_ = 3.07 min, 85 A% @ 254 nm, [M + H]^+^ = 172.1. ^1^H NMR (600 MHz, DMSO-*d_6_*) δ 7.92 (s, 1H), 4.09 (s, 3H).

N-(4-fluorobenzyl)-1-methyl-5-nitro-1H-imidazole-2-carboxamide; **8a**

**

General procedure A. Amine 4-fluorobenzylamine (72 µL, 0.63 mmol). The crude residue was purified by MPLC over silica gel (Biotage, 6–50% EtOAc in pet. spirits gradient) to yield a yellow solid (85 mg, 58%). LCMS: R_t_ = 3.49 min, 99 A% @ 254 nm, [M - H]^-^ = 277.0. ^1^H NMR (600 MHz, DMSO-*d*_6_) δ 9.56 (t, *J* = 6.4 Hz, 1H), 8.18 (s, 1H), 7.40 – 7.33 (m, 2H), 7.21 – 7.12 (m, 2H), 4.42 (d, *J* = 6.3 Hz, 2H), 4.26 (s, 3H). ^13^C NMR (150 MHz, DMSO-*d*_6_) δ 161.1 (d, *J* = 241.7 Hz), 157.8, 141.1, 140.6, 135.0 (d, *J* = 3.0 Hz), 130.9 (d, *J* = 1.8 Hz), 129.4 (d, *J* = 8.0 Hz), 115.0 (d, *J* = 21.4 Hz), 41.5, 34.5. HRMS (ESI): *m/z* calcd for C_12_H_11_FN_4_NaO_3_ [M + Na]^+^, 301.0707; found, 301.0701.

1-methyl-5-nitro-N-(4-(trifluoromethoxy)benzyl)-1H-imidazole-2-carboxamide; **8b**

**

General procedure A. Amine: 4-(trifluoromethoxy)benzylamine (97 µL, 0.63 mmol). The crude residue was purified by MPLC over silica gel (Biotage, 6–50% EtOAc in pet. spirits gradient then repurified with 0–2% MeOH in DCM gradient) to yield a yellow solid (26 mg, 14%). LCMS: R_t_ = 3.69 min, 99 A% @ 254 nm, [M - H]^-^ = 342.9. ^1^H NMR (600 MHz, DMSO-*d*_6_) δ 9.61 (t, *J* = 6.4 Hz, 1H), 8.18 (s, 1H), 7.47 – 7.42 (m, 2H), 7.36 – 7.30 (m, 2H), 4.46 (d, *J* = 6.3 Hz, 2H), 4.25 (s, 3H). ^13^C NMR (150 MHz, DMSO-*d*_6_) δ 157.9, 147.1 (q, *J* = 1.8 Hz), 140.9, 140.6, 138.3, 130.9, 129.1, 120.9, 120.0 (q, *J* = 255.8 Hz), 41.5, 34.5. HRMS (ESI): *m/z* calcd for C_13_H_12_F_3_N_4_O_4_ [M + H]^+^, 345.0805; found, 345.0802.

1-methyl-5-nitro-N-(3-(trifluoromethoxy)benzyl)-1H-imidazole-2-carboxamide; **8c**

**

General procedure A. Amine: 3-(trifluoromethoxy)benzylamine (129 µL, 0.63 mmol). The crude residue was purified by MPLC over silica gel (Biotage, 6–50% EtOAc in pet. spirits gradient) to yield a yellow oil (100 mg, 55%). LCMS: R_t_ = 3.70 min, 99 A% @ 254 nm, [M + H]^+^ = 345.0. ^1^H NMR (600 MHz, DMSO-*d*_6_) δ 9.63 (t, *J* = 6.4 Hz, 1H), 8.19 (s, 1H), 7.47 (t, *J* = 7.9 Hz, 1H), 7.39 – 7.34 (m, 1H), 7.32 (s, 1H), 7.28 – 7.23 (m, 1H), 4.49 (d, *J* = 6.4 Hz, 2H), 4.26 (s, 3H). ^13^C NMR (150 MHz, DMSO-*d*_6_) δ 157.9, 148.3 (q, *J* = 1.8 Hz), 141.6, 141.0, 140.6, 130.9, 130.2, 126.3, 120.0 (q, *J* = 256.5 Hz), 119.6, 119.3, 41.7, 34.5. HRMS (ESI): *m/z* calcd for C_13_H_12_F_3_N_4_O_4_ [M + H]^+^, 345.0805; found, 345.0802.

N-(1-(4-fluorophenyl)ethyl)-1-methyl-5-nitro-1H-imidazole-2-carboxamide; **8d**

**

General Procedure A. Amine: 1-(4-fluorophenyl)ethan-1-amine (92 µL, 0.68 mmol). The crude residue was purified by MPLC over silica gel (Biotage, 6–50% EtOAc in pet. spirits gradient) to yield a yellow solid (89 mg, 54%). LCMS: R_t_ = 3.57 min, 99 A% @ 254 nm, [M - H]^-^ = 290.9*.* ^1^H NMR (600 MHz, DMSO-*d*_6_) δ 9.40 (d, *J* = 8.4 Hz, 1H), 8.17 (s, 1H), 7.50 – 7.42 (m, 2H), 7.19 – 7.12 (m, 2H), 5.14 (p, *J* = 7.2 Hz, 1H), 4.21 (s, 3H), 1.49 (d, *J* = 7.1 Hz, 3H). ^13^C NMR (150 MHz, DMSO-*d*_6_) δ 161.0 (d, *J* = 243.5 Hz), 157.0, 141.2, 140.5, 140.1 (d, *J* = 2.9 Hz), 130.8 (d, *J* = 1.9 Hz), 128.1 (d, *J* = 8.0 Hz), 114.9 (d, *J* = 20.4 Hz), 47.7, 34.4, 21.6. HRMS (ESI): *m/z* calcd for C_13_H_14_FN_4_O_3_ [M + H]^+^, 293.1044; found, 293.1048.

1-methyl-N-(4-methylphenethyl)-5-nitro-1H-imidazole-2-carboxamide; **8e**

**

General procedure A. Amine: 2-(p-tolyl)ethan-1-amine (92 µL, 0.63 mmol). The crude residue was purified by MPLC over silica gel (Biotage, 6–50% EtOAc in pet. spirits gradient) to yield a yellow solid (60 mg, 39%). LCMS: R_t_ = 3.64 min, 99 A% @ 254 nm, [M + H]^+^ = 289.1. ^1^H NMR (600 MHz, CDCl_3_) δ 7.93 (s, 1H), 7.60 (br t, *J* = 6.1 Hz, 1H), 7.18 – 7.10 (m, 4H), 4.47 (s, 3H), 3.67 (q, *J* = 7.1 Hz, 2H), 2.90 (t, *J* = 7.1 Hz, 2H), 2.35 (s, 3H). ^13^C NMR (150 MHz, CDCl_3_) δ 157.8, 140.8, 140.6, 136.3, 135.1, 131.0, 129.4, 128.6, 40.8, 35.2, 35.0, 21.1. HRMS (ESI): *m/z* calcd for C_14_H_16_N_4_NaO_3_ [M + Na]^+^, 311.1115; found, 311.1116.

1-methyl-5-nitro-N-(pyridin-2-ylmethyl)-1H-imidazole-2-carboxamide.xTFA; **8f**

**

General procedure A. Amine: pyridin-2-ylmethanamine (66 µL, 0.68 mmol). The crude residue was purified by MPLC over C18 silica gel (Grace Reveleris X2, A: H_2_O + 0.1% TFA, B: ACN + 0.1% TFA, 5–25% B). The eluent was removed by lyophilisation to yield a colourless solid (59 mg, 32%). LCMS: R_t_ = 2.61 min, 99 A% @ 254 nm, [M + H]^+^ = 262*.*0. ^1^H NMR (600 MHz, DMSO-*d*_6_) δ 9.56 (t, *J* = 6.0 Hz, 1H), 8.63 (d, *J* = 5.1 Hz, 1H), 8.21 (s, 1H), 8.00 (t, *J* = 7.9 Hz, 1H), 7.54 (d, *J* = 8.0 Hz, 1H), 7.49 (t, *J* = 6.5 Hz, 1H), 4.65 (d, *J* = 6.0 Hz, 2H), 4.27 (s, 3H). ^13^C NMR (150 MHz, DMSO-*d*_6_) δ 158.1, 156.1, 146.4, 140.8, 140.6, 139.1, 131.0, 123.2, 123.2, 42.9, 34.5. HRMS (ESI): *m/z* calcd for C_11_H_12_N_5_O_3_ [M + H]^+^, 262.0935; found, 262.0943.

N,N,1-trimethyl-5-nitro-1H-imidazole-2-carboxamide; **8g**

General procedure A. Amine: 2M dimethyl amine in THF (391 µL, 0.78 mmol). The crude residue was purified by MPLC over silica gel (Biotage, 25–100% EtOAc in pet. spirits gradient) to yield a yellow solid (27 mg, 41%). LCMS: R_t_ = 2.56 min, 99 A% @ 254 nm, [M + H]^+^ = 199*.*1. ^1^H NMR (600 MHz, CDCl_3_) δ 7.98 (s, 1H), 4.11 (s, 3H), 3.23 (s, 3H), 3.15 (s, 3H). ^13^C NMR (150 MHz, CDCl_3_) δ 159.3, 143.6, 139.5, 131.1, 38.9, 35.7, 35.0. HRMS (ESI): *m/z* calcd for C_7_H_10_N_4_NaO_3_ [M + Na]^+^, 221.0645; found, 221.0644.

(1-methyl-5-nitro-1H-imidazol-2-yl)(morpholino)methanone; **8h**

**

General procedure A except the compound was extracted with DCM (6 x 100 vol). Amine: morpholine (68 µL, 0.78 mmol). The crude residue was purified by MPLC over silica gel (Biotage, 50–100% EtOAc in pet. spirits gradient) to yield a yellow solid (34 mg, 44%). LCMS: R_t_ = 2.69 min, 99 A% @ 254 nm, [M + H]^+^ = 241.0. ^1^H NMR (600 MHz, CDCl_3_) δ 7.97 (s, 1H), 4.13 (s, 3H), 3.87 – 3.83 – 3.82 (m, 2H), 3.81 (br s, 4H), 3.78 – 3.73 (m, 2H). ^13^C NMR (150 MHz, CDCl_3_) δ 157.8, 142.8, 139.8, 130.9, 67.0, 66.7, 47.8, 42.9, 35.0. HRMS (ESI): *m/z* calcd for C_9_H_12_N_4_NaO_4_ [M + Na]^+^, 263.0751; found, 263.0762.

(1-methyl-5-nitro-1H-imidazol-2-yl)(pyrrolidin-1-yl)methanone; **8i**

**

General procedure A. Amine: pyrrolidine (56 µL, 0.68 mmol). The crude residue was purified by MPLC over silica gel (Biotage, 12–100% EtOAc in pet. spirits gradient) to yield a yellow solid (54 mg, 43%). LCMS: R_t_ = 2.90 min, 99 A% @ 254 nm, [M + H]^+^ = 225.0. ^1^H NMR (600 MHz, CDCl_3_) δ 7.97 (s, 1H), 4.22 (s, 3H), 3.84 – 3.81 (m, 2H), 3.67 – 3.65 (m, 2H), 1.99– 1.96 (m, 4H). ^13^C NMR (150 MHz, CDCl_3_) δ 157.2, 143.7, 139.7, 131.0, 49.1, 46.9, 35.1, 26.3, 23.9. HRMS (ESI): *m/z* calcd for C_9_H_12_N_4_NaO_3_ [M + Na]^+^, 247.0802; found, 247.0808.

N-cyclopropyl-1-methyl-5-nitro-1H-imidazole-2-carboxamide; **8j**

General procedure A. Amine: cyclopropylamine (47 µL, 0.68 mmol). The crude residue was purified by MPLC over silica gel (Biotage, 6–50% EtOAc in pet. spirits gradient) to yield a yellow solid (57 mg, 48%). LCMS: R_t_ = 2.93 min, 99 A% @ 254 nm, [M + H]^+^ = 211.1. ^1^H NMR (600 MHz, CDCl_3_) δ 7.91 (s, 1H), 7.58 (s, 1H), 4.47 (s, 3H), 2.89 – 2.85 (m, 1H), 0.95 – 0.85 (m, 2H), 0.70 – 0.63 (m, 2H). ^13^C NMR (150 MHz, DMSO-*d*_6_) δ 158.9, 141.3, 140.5, 130.8, 40.3, 34.4, 22.8, 5.5. HRMS (ESI): *m/z* calcd for C_8_H_10_N_4_NaO_3_ [M + Na]^+^, 233.0645; found, 233.0652.

N-cyclohexyl-1-methyl-5-nitro-1H-imidazole-2-carboxamide; **8k**

General procedure A. Amine: cyclohexylamine (78 µL, 0.68 mmol). The crude residue was purified by MPLC over silica gel (Biotage, 3–40% EtOAc in pet. spirits gradient) to yield a yellow solid (63 mg, 44%). LCMS: R_t_ = 3.51 min, 99 A% @ 254 nm, [M + H]^+^ = 253.1. ^1^H NMR (600 MHz, CDCl_3_) δ 7.95 (s, 1H), 7.44 (d, *J* = 5.3 Hz, 1H), 4.48 (s, 3H), 3.96 – 3.86 (m, 1H), 2.03 – 1.86 (m, 2H), 1.83 – 1.72 (m, 2H), 1.71 – 1.64 (m, 1H), 1.49 – 1.36 (m, 2H), 1.36 – 1.20 (m, 3H). ^13^C NMR (150 MHz, CDCl_3_) δ 156.9, 141.1, 140.6, 130.9, 48.6, 35.1, 32.8, 25.4, 24.8. HRMS (ESI): *m/z* calcd for C_11_H_16_N_4_NaO_3_ [M + Na]^+^, 275.1115; found, 275.1122.

5-nitro-1H-imidazole-2-carboxylic acid; **10**

Conc. H_2_SO_4_ (27.5 mL, 516 mmol) was added slowly to 1H-imidazole-2-carboxylic acid (5 g, 44.6 mmol) followed by drop wise addition of fuming HNO_3_ (5 mL). The reaction was then heated to 80 °C for 5.5 hr. The reaction was cooled to rt, then added drop wise over crushed ice. The precipitate was then filtered and dried under vacuum overnight to yield a pale lemon solid (3.8 g, 54%). LCMS (Waters Atlantis: Gradient Method 1): R_t_ = 1.35 min, 99 A% @ 254 nm, [M + H]^+^ = 158.0. ^1^H NMR (400 MHz, DMSO δ 14.38 (s, 1H), 8.48 (s, 1H). ^13^C NMR (150 MHz, CDCl_3_) δ 158.8, 147.3, 136.8, 121.6. HRMS (ESI): *m/z* calcd for C_8_H_6_N_6_NaO_8_ [2M + Na]^+^, 337.0139; found, 337.0151.

N-(4-fluorobenzyl)-5-nitro-1H-imidazole-2-carboxamide; **12a**

General procedure B. Amine: (4-fluorophenyl)methanamine (330 mg, 2.63 mmol). The crude was purified by MPLC over silica gel (Biotage, 15–100% EtOAc in pet. spirits gradient, then repurified with 6–60% gradient) to yield a cream solid (411 mg, 59%). LCMS: R_t_ = 3.21 min, 99 A% @ 254 nm, [M - H]^-^ = 262.9. ^1^H NMR (600 MHz, DMSO-*d*_6_) δ 14.30 (s, 1H), 9.46 (t, *J* = 6.4 Hz, 1H), 8.46 (s, 1H), 7.40 – 7.33 (m, 2H), 7.18 – 7.11 (m, 2H), 4.41 (d, *J* = 6.3 Hz, 2H). ^13^C NMR (150 MHz, DMSO-*d*_6_) δ 161.1 (d, *J* = 232 Hz), 157.1, 146.6, 139.6, 135.1 (d, *J* = 3.1 Hz), 129.4 (d, *J* = 8.6 Hz), 121.5, 115.4 (d, *J* = 21.7 Hz), 41.5. HRMS (ESI): *m/z* calcd for C_11_H_9_F_1_N_4_NaO_3_ [M + Na]^+^, 287.0551; found, 287.0556.

5-nitro-N-(4-(trifluoromethoxy)benzyl)-1H-imidazole-2-carboxamide; **12b**

General procedure B. Amine: (4-trifluoromethoxy)phenyl)methanamine (408 µL, 2.67 mmol). The crude was purified by MPLC over silica gel (Biotage, 30–100% EtOAc in pet. spirits gradient) to yield a colourless solid (841 mg, 75 %). LCMS: R_t_ = 3.46 min, 99 A% @ 254 nm, [M + H]^+^ = 328.9. ^1^H NMR (600 MHz, DMSO-*d*_6_) δ 14.33 (s, 1H), 9.53 (t, *J* = 6.3 Hz, 1H), 8.47 (s, 1H), 7.45 (d, *J* = 8.7 Hz, 2H), 7.32 (d, *J* = 8.2 Hz, 2H), 4.46 (d, *J* = 6.3 Hz, 2H). ^13^C NMR (150 MHz, DMSO-*d*_6_) δ 157.2, 147.1 (q, *J* = 1.8 Hz), 146.7, 139.5, 138.5, 129.1, 121.6, 120.9, 120.0 (q, *J* = 255.0 Hz), 41.5. HRMS (ESI): *m/z* calcd for C_12_H_9_F_3_N_4_NaO_4_ [M + Na]^+^, 353.0468; found, 353.0462.

5-nitro-N-(3-(trifluoromethoxy)benzyl)-1H-imidazole-2-carboxamide; **12c**

General procedure C. Amine: 3-(trifluoromethoxy)benzylamine (263 mg, 1.37 mmol). The crude material was purified by MPLC over C18 silica gel (Grace Reveleris X2, A: H_2_O + 0.1% TFA, B: ACN + 0.1% TFA, 30–100% B). The eluent was removed by lyophilisation to yield a colourless solid (259 mg, 60%). LCMS: R_t_ = 3.43 min, 99 A% @ 254 nm, [M + H]^+^ = 331.0. ^1^H NMR (600 MHz, DMSO-*d*_6_) δ 14.32 (s, 1H), 9.54 (t, *J* = 6.4 Hz, 1H), 8.47 (s, 1H), 7.47 (t, *J* = 7.9 Hz, 1H), 7.36 (dt, *J* = 7.8, 1.2 Hz, 1H), 7.31 (s, 1H), 7.25 (ddt, *J* = 1.1, 2.5, 8.3 Hz, 1H), 4.48 (d, *J* = 6.4 Hz, 2H). ^13^C NMR (150 MHz, DMSO-*d*_6_) δ 157.2, 148.2 (q, *J* = 1.7 Hz), 146.7, 141.8, 139.4, 130.2, 126.4, 121.5, 120.0 (q, *J* = 255 Hz), 119.7, 119.3, 41.7. HRMS (ESI): *m/z* calcd for C_24_H_18_F_6_N_8_NaO_8_ [2M + Na]^+^, 683.1044; found, 683.1025.

N-(1-(4-fluorophenyl)ethyl)-5-nitro-1H-imidazole-2-carboxamide; **12d**

General procedure C. Amine: 4-fluoro-alpha-methylbenzylamine (180 µL, 1.37 mmol). The crude material was purified by MPLC over silica gel (Grace Reveleris X2, 20–100% EtOAc in pet. spirits gradient) to yield a yellow solid (224 mg, 74 %). LCMS: R_t_ = 3.29 min, 99 A% @ 254 nm, [M - H]- = 277.0. ^1^H NMR (600 MHz, DMSO-*d*_6_) δ 14.23 (s, 1H), 9.31 (d, *J* = 8.5 Hz, 1H), 8.46 (d, *J* = 1.5 Hz, 1H), 7.50 – 7.41 (m, 2H), 7.17 – 7.10 (m, 2H), 5.14 (p, *J* = 7.2 Hz, 1H), 1.50 (d, *J* = 7.1 Hz, 3H). ^13^C NMR (150 MHz, DMSO-*d*_6_) δ 161.5 (d, *J* = 243.2 Hz), 156.8, 147.2, 140.8 (d, *J* = 3.0 Hz), 140.0, 128.7 (d, *J* = 8.3 Hz), 122.0, 115.4 (d, *J* = 21.5 Hz), 48.2, 22.1. HRMS (ESI): *m/z* calcd for C_12_H_11_FN_4_NaO_3_ [M + Na]^+^, 301.0707; found, 301.0703.

N-(4-methylphenethyl)-5-nitro-1H-imidazole-2-carboxamide; **12e**

**

General procedure C. Amine: 2-(p-tolyl)ethan-1-amine (300 µL, 2.07 mmol). The crude material was purified by MPLC over silica gel (Biotage 20–100% EtOAc in pet. spirits gradient, then 0–5% MeOH) to yield an orange solid (167 mg, 35%). LCMS: R_t_ = 3.37 min, 98 A% @ 254 nm, [M + H]^+^ = 275.0. ^1^H NMR (600 MHz, DMSO-*d*_6_) δ δ 14.27 (s, 1H), 8.90 (t, *J* = 6.0 Hz, 1H), 8.43 (s, 1H), 7.12 – 7.08 (m, 4H), 3.45 (dt, *J* = 7.9, 6.2 Hz, 2H), 2.79 (t, *J* = 7.4 Hz, 2H), 2.25 (s, 3H). ^13^C NMR (150 MHz, DMSO-*d*_6_) δ 157.5, 146.7, 139.9, 136.1, 135.0, 128.9, 128.5, 121.6, 40.3, 34.5, 20.6.HRMS (ESI): *m/z* calcd for C_26_H_28_N_8_NaO_6_ [2M + Na]^+^, 571.2024; found, 571.2032.

5-nitro-N-(pyridin-2-ylmethyl)-1H-imidazole-2-carboxamide; **12f**

General procedure C except when the reaction was completed the volatiles were removed *in vacuo* to obtain a crude residue. The crude was suspended in MeOH/EtOAc and evaporated onto C18 silica gel and purified by MPLC (Grace Reveleris X2, A: H_2_O + 0.1% TFA, B: ACN + 0.1% TFA, 5–20% B) to yield a yellow solid (263 mg, 93%). Amine: 2-picolylamine (141 µL, 1.37 mmol). LCMS: R_t_ = 2.20 min, 99 A% @ 254 nm, [M + H]^+^ = 248.1. ^1^H NMR (600 MHz, DMSO-*d*_6_) δ 14.38 (s, 1H), 9.48 (t, *J* = 6.1 Hz, 1H), 8.64 – 8.60 (m, 1H), 8.50 (s, 1H), 8.01 (td, *J* = 7.8, 1.8 Hz, 1H), 7.53 (d, *J* = 7.9 Hz, 1H), 7.49 (dd, *J* = 7.3, 5.5 Hz, 1H), 4.64 (s, 1H). ^13^C NMR (150 MHz, DMSO-*d*_6_) δ 157.4, 156.3, 146.6, 146.4, 139.7, 139.2, 123.1, 122.2, 121.6, 42.8. HRMS (ESI): *m/z* calcd for C_10_H_10_N_5_O_3_ [M + H]^+^, 248.0778; found, 248.0781.

N,N-dimethyl-5-nitro-1H-imidazole-2-carboxamide; **12g**

General procedure B. Amine: dimethylamine in THF 2.0 M (525 µL, 1.05 mmol). The reaction was poured into water (60 mL) and extracted with DCM (50 mL) to remove the phosphoryl oxide byproduct. The aq. layer was then acidified to pH 4.0 with aq. HCl. The aq. layer was then extracted with DCM (3 x 50 mL). The combined organic layers were washed with brine (40 mL), dried with MgSO_4_, filtered and volatiles removed in vacuo. The crude material was purified by MPLC over silica gel (Biotage 80–100 % EtOAc in pet. spirit gradient, followed by MeOH 0–10% gradient), slurried in MeOH and then purified by MPLC over C18 silica gel (Reveleris X2 A: H_2_O + 0.1% TFA, B: ACN + 0.1% TFA, 5–80% B) to yield colourless solid (29 mg, 16%). LCMS: R_t_ = 2.47 min, 99 A% @ 254 nm, [M + H]^+^ = 185.0. ^1^H NMR (600 MHz, DMSO-*d*_6_) δ 14.10 (s, 1H), 8.43 (s, 1H), 3.49 (s, 3H), 3.03 (s, 3H). ^13^C NMR (150 MHz, DMSO-*d*_6_) δ 158.2, 146.7, 140.0, 121.2, 38.6, 36.6. HRMS (ESI): *m/z* calcd for C_6_H_8_N_4_NaO_3_ [M + Na]^+^, 207.0489; found, 207.0481.

Morpholino(5-nitro-1H-imidazol-2-yl)methanone; **12h**

******

General procedure B. Amine: morpholine (92 µL, 1.05 mmol). The reaction was poured into water (60 mL) and extracted with EtOAc (50 mL) to remove the phosphoryl oxide byproduct. The aq. layer was then acidified to pH 4.0 with aq. HCl. The aq. layer was extracted with EtOAc (1 x 50mL). Brine (10 mL) was added to aid layer separation. The aq. layer was then extracted with EtOAc (2 x 50 mL). The combined organic layers were washed with brine (50 mL), dried with MgSO_4_, filtered and volatiles removed in vacuo. The crude material was then purified by MPLC over c18 silica gel (Reveleris X2 A: H_2_O + 0.1% TFA, B: ACN + 0.1% TFA, 5–100% B) to yield a colourless powder (15 mg, 6%). LCMS: R_t_ = 2.57 min, 99 A% @ 254 nm, [M + H]^+^ = 227.0. ^1^H NMR (600 MHz, DMSO-*d*_6_) δ 14.18 (s, 1H), 8.45 (s, 1H), 4.30 (t, *J* = 4.8 Hz, 2H), 3.69 – 3.62 (m, 6H). ^13^C NMR (150 MHz, DMSO-*d*_6_) δ 156.3, 146.2, 139.1, 120.9, 66.3, 66.1, 46.7, 42.7. HRMS (ESI): *m/z* calcd for C_8_H_10_N_4_NaO_4_ [M + Na]^+^, 249.0594; found, 249.0599.

(5-nitro-1H-imidazol-2-yl)(pyrrolidin-1-yl)methanone; **12i**

******

General procedure B. Amine: ethanolamine (58 µL, 1.10 mmol). The reaction was poured into water (60 mL) and extracted with EtOAc (50 mL) to remove the phosphoryl oxide byproduct. The aq. Layer was then acidified to pH 4.0 with aq. HCl. The aq. layer was then extracted with EtOAc (3 x 50 mL). The combined organic layers were washed with brine (50 mL), dried with MgSO_4_, filtered and volatiles removed in vacuo. The crude material was then purified by MPLC over C18 silica gel (Reveleris X2 A: H_2_O + 0.1% TFA, B: ACN + 0.1% TFA, 5–80% B) to yield the target product 1 and product 2, a pyrrolidine impurity formed by coupling pyrrolidine from degraded PyBOP with the carboxylic acid [3]. **Product 1**: N-(2-hydroxyethyl)-5-nitro-1H-imidazole-2-carboxamide as a white solid (46 mg). LCMS: R_t_ = 1.89 min, 99 A% @ 254 nm, [M + H]^+^ = 201.2. Product 1 was contaminated with DIPEA (^1^H NMR) and was unsuitable for biological assay and was prepared instead via the acid chloride intermediate (**12n**). **Product 2 (12i)**: (5-nitro-1H-imidazol-2-yl)(pyrrolidin-1-yl)methanone as a white solid (19 mg, 10%). LCMS: R_t_ = 2.85 min, 99 A% @ 254 nm, [M + H]^+^ = 211.1. ^1^H NMR (600 MHz, DMSO-*d*_6_) δ 14.15 (s, 1H), 8.43 (s, 1H), 3.99 (t, *J* = 6.8 Hz, 2H), 3.52 (t, *J* = 6.9 Hz, 2H), 1.94 (p, *J* = 6.8 Hz, 2H), 1.84 (p, *J* = 6.8 Hz, 2H). ^13^C NMR (150 MHz, DMSO-*d*_6_) δ 155.8, 146.7, 140.0, 120.9, 48.5, 47.0, 26.0, 23.3. HRMS (ESI): *m/z* calcd for C_8_H_10_N_4_NaO_3_ [M + Na]^+^, 233.0645; found, 233.0644.

N-cyclopropyl-5-nitro-1H-imidazole-2-carboxamide; **12j**

General procedure C. Amine: cyclopropylamine (94 µL, 1.37 mmol). The crude material was purified by MPLC over silica gel (Grace Reveleris X2, 20–100% EtOAc in pet. spirits gradient) then repurified over C18 silica gel (Grace Reveleris X2, A: H_2_O + 0.1% TFA, B: ACN + 0.1% TFA, 5–100% B) to yield a yellow solid (32 mg, 14%). LCMS: R_t_ = 2.58 min, 99 A% @ 254 nm, [M + H]^+^ = 197.0. ^1^H NMR (600 MHz, DMSO-*d*_6_) δ 14.25 (s, 1H), 8.91 (d, *J* = 4.9 Hz, 1H), 8.43 (s, 1H), 2.89 – 2.85 (m, 1H), 0.68 – 0.65 (m, 4H). ^13^C NMR (150 MHz, DMSO-*d*_6_) δ 158.3, 146.7, 139.7, 121.4, 22.9, 5.6. HRMS (ESI): *m/z* calcd for C_7_H_8_N_4_NaO_3_ [M + Na]^+^, 219.0489; found, 219.0489.

N-cyclohexyl-5-nitro-1H-imidazole-2-carboxamide; **12k**

General procedure C. Amine: cyclohexylamine (156 µL, 1.37 mmol). The crude material was purified by MPLC over silica gel (Grace Reveleris X2, 20–100% EtOAc in pet. spirits gradient) to obtain a yellow solid (122 mg, 45%). LCMS: R_t_ = 3.18 min, 99 A% @ 254 nm, [M + H]^+^ = 239.0. ^1^H NMR (600 MHz, DMSO-*d*_6_) δ 14.25 (s, 1H), 8.43 (d, *J* = 8.6 Hz, 1H), 8.30 (s, 1H), 3.77 – 3.70 (m, 1H), 1.76 – 1.70 (m, 5H), 1.60 – 1.58 (m, 1H), 1.45 – 1.39 (m, 2H), 1.34 – 1.22 (m, 2H), 1.14 – 1.07 (m, 1H). ^13^C NMR (150 MHz, DMSO-*d*_6_) δ 156.7, 146.9, 140.9, 122.5, 48.1, 32.0, 25.1, 25.0. HRMS (ESI): *m/z* calcd for C_10_H_14_N_4_NaO_3_ [M + Na]^+^, 261.0958; found, 261.0966.

5-nitro-1H-imidazole-2-carboxamide; **12l**

General procedure C to form the acid chloride. The acid chloride (1.29 g, 7.35 mmol) was then added portionwise into conc. NH_4_OH (38 mL) at 0 °C [caution: significant gas evolution (NH_3_)]. Volatiles were removed in vacuo to yield a yellow solid (1.24 g, 92%) that was of sufficient purity for further synthesis. A 100 mg sample of crude was purified by MPLC over C18 silica gel (Grace Reveleris X2, A: H_2_O + 0.1% TFA, B: ACN + 0.1% TFA, 5–30% B) to yield a colourless solid (39 mg). LCMS: R_t_ = 1.65 min, 99 A% @ 254 nm, [M + H]^+^ = 157.3. ^1^H NMR (600 MHz, DMSO-*d*_6_) δ 14.24 (s, 1H), 8.44 (s, 1H), 8.23 (s, 1H), 7.83 (s, 1H). ^13^C NMR (150 MHz, DMSO-*d*_6_) δ 158.9, 146.7, 139.9, 121.5. HRMS (ESI): *m/z* calcd for C_4_H_4_N_4_NaO_3_ [M + Na]^+^, 179.0176; found, 179.0172.

N-methyl-5-nitro-1H-imidazole-2-carboxamide; **12m**

General procedure C (amine: methylamine hydrochloride (365 mg, 5.47 mmol)) except when the reaction was complete the volatiles were removed *in vacuo* to obtain the crude residue. The crude material was suspended in water, acidified with 2M aq. HCl and the product obtained by filtration. The solid was washed with water portions to remove residual acid. The crude material was obtained as a tan solid that contained residual TEA (289 mg, 37%). A 100 mg portion of crude material was purified by MPLC over C18 silica gel (Grace Reveleris X2, A: H_2_O + 0.1% TFA, B: ACN + 0.1% TFA, 30–100% B) to yield a colourless solid (30 mg). LCMS: R_t_ = 2.17 min, 99 A% @ 254 nm, [M + H]^+^ = 171.1. ^1^H NMR (600 MHz, DMSO-*d*_6_) δ 14.26 (s, 1H), 8.83 (d, *J* = 5.2 Hz, 1H), 8.43 (s, 1H), 2.77 (d, *J* = 4.7 Hz, 3H). ^13^C NMR (150 MHz, DMSO-*d*_6_) δ 157.5, 146.7, 139.8, 121.3, 25.9.. HRMS (ESI): *m/z* calcd for C_5_H_6_N_4_NaO_3_ [M + Na]^+^, 193.0332; found, 193.0340.

N-(2-hydroxyethyl)-5-nitro-1H-imidazole-2-carboxamide; **12n**

General procedure C (amine: ethanolamine (82 µL, 1.37 mmol)) except when the reaction was complete the volatiles were removed *in vacuo* to afford a crude residue. The isolated solid (170 mg) contained TEA which was removed by suspending the solid in H_2_O (4 mL) and acidifying with 2M aq. HCl. The solid was then isolated by filtration and washed with water (3 x 250 µL). The solid was dried *in vacuo* to yield a colourless solid (105 mg, 46%). LCMS: R_t_ = 1.89 min, 99 A% @ 254 nm, [M + H]^+^ = 201.2. ^1^H NMR (600 MHz, DMSO-*d*_6_) δ 14.28 (s, 1H), 8.68 (t, *J* = 5.9 Hz, 1H), 8.44 (s, 1H), 4.76 (t, *J* = 5.7 Hz, 1H), 3.50 (q, *J* = 6.0 Hz, 2H), 3.32* (q, *J* = 6.3 Hz, 2H). ^13^C NMR (150 MHz, DMSO-*d*_6_) δ 157.2, 146.7, 139.8, 121.5, 59.4, 41.6. HRMS (ESI): *m/z* calcd for C_6_H_8_N_4_NaO_4_ [M + Na]^+^, 223.0438; found, 223.0430. *coincident with H_2_O signal that precluded accurate integration.

N-(2-hydroxyethyl)-N-methyl-5-nitro-1H-imidazole-2-carboxamide; **12o**

General procedure C. Amine: N-Methylethanolamine (110 µL, 1.37 mmol). The crude material was purified by MPLC over C18 silica gel (Grace Reveleris X2, A: H_2_O + 0.1% TFA, B: ACN + 0.1% TFA, 5–100% B) to obtain a colourless solid (30 mg, 12%). LCMS: R_t_ = 2.28 min, 99 A% @ 254 nm, [M + H]^+^ = 215.0. Rotamers were observed in the ^1^H NMR (0.73 (minor): 1 (major) ratio) and ^13^C NMR. ^1^H NMR (600 MHz, DMSO-*d*_6_) δ 14.07 (s, 1H), 8.43 (min) and 8.41 (maj) (rotamers, s, 1H), 4.81 (min/maj indistinguishable) (rotamer, t, *J* = 5.5 Hz, 1H), 4.74 (min/maj indistinguishable) (rotamer, br s, 1H), 4.05 (maj) and 3.53 (min) (rotamers, t, *J* = 5.7 Hz, 2H), 3.60 (t, *J* = 5.5 Hz, 2H), 3.52 (min) and 3.04 (maj) (s, 3H). ^13^C NMR (150 MHz, DMSO-*d*_6_) δ 158.4 (maj), 157.6 (min), 146.2 (min), 146.0 (maj), 139.7 (maj), 139.6 (min), 120.6 (min), 120.5 (maj), 58.7 (maj), 58.0 (min), 51.8 (maj), 51.0 (min), 37.6 (min), 34.6 (maj). HRMS (ESI): *m/z* calcd for C_7_H_10_N_4_NaO_4_ [M + Na]^+^, 237.0594; found, 237.0602.

Ethyl 5-nitro-1H-imidazole-2-carboxylate; **12p**

An ice-cold solution of ethylimidazole-2-carboxylate (5.1 g, 36.4 mmol) in conc sulfuric acid (15 mL, 281 mmol) was treated dropwise with fuming HNO_3_ (15 mL). The mixture was heated at 60 °C for 2.5 hr, cooled and then poured onto ice (150 g). The resulting solid was collected by vacuum filtration, washed with water, and dried *in vacuo* to afford the title compound **12p** as a colourless powder (4.2 g, 64%). LCMS: R_t_ = 2.49 min, 99 A% @ 254 nm, [M - H]^-^ = 184.0. ^1^H NMR (600 MHz, DMSO-*d*_6_) δ 8.55 (s, 1H), 4.38 (q, *J* = 7.1 Hz, 2H), 1.35 (t, *J* = 7.1 Hz, 3H). ^13^C NMR (150 MHz, DMSO-*d*_6_) δ 157.6, 147.4, 135.9, 122.1, 61.9, 14.0. The ^1^H data was consistent with previous reports [4].

1-(4-fluorobenzyl)-4-nitro-1H-imidazole-2-carboxamide; **13a**

General procedure C, rt, isolated by precipitation. Benzyl bromide: 1-(bromomethyl)-4 fluorobenzene (120 µL, 0.96 mol). The crude product was purified by recrystallization (EtOH) to yield colourless crystals (62 mg, 29%). Mp = 202–204 °C. LCMS: R_t_ = 3.22 min, 99 A% @ 254 nm, [M + H]^+^ = 265.1. ^1^H NMR (600 MHz, DMSO-*d*_6_) δ 8.74 (s, 1H), 8.21 (s, 1H), 7.85 (s, 1H), 7.45 – 7.41 (m, 2H), 7.22 – 7.17 (m, 2H), 5.70 (s, 2H).^13^C NMR (150 MHz, DMSO-*d*_6_) δ 161.8 (d, *J* = 245.7 Hz), 159.4, 144.8, 137.0, 132.8, 130.1 (d, *J* = 8.2 Hz), 125.5, 115.6 (d, *J* = 21.8 Hz), 50.7. HRMS (ESI): *m/z* calcd for C_12_H_10_F_1_N_4_NaO_3_ [M + Na]^+^, 287.0551; found, 287.0554.

4-nitro-1-(4-(trifluoromethoxy)benzyl)-1H-imidazole-2-carboxamide; **13b**

General procedure C, rt, isolated by precipitation. Benzyl bromide: 1-(bromomethyl)-4-(trifluoromethoxy)benzene (186 µL, 1.15 mmol). The crude product was purified by recrystallization (EtOH) to yield cream coloured crystals (78 mg, 25 %). Mp = 204–205 °C. LCMS: R_t_ = 3.47 min, 99 A% @ 254 nm, [M - H]^-^ = 328.9. ^1^H NMR (600 MHz, DMSO-*d*_6_) δ 8.76 (s, 1H), 8.21 (br s, 1H), 7.84 (br s, 1H), 7.48 – 7.45 (m, 2H), 7.38 – 7.35 (m, 2H), 5.75 (s, 2H). ^13^C NMR (150 MHz, DMSO-*d*_6_) δ 159.3, 147.8 (q, *J* = 1.8 Hz), 144.8, 137.0, 135.9, 129.6, 125.5, 121.2, 120.0 (q, *J* = 253.2 Hz), 50.6. HRMS obtained from previous preparation. HRMS (ESI): *m/z* calcd for C_12_H_9_F_3_N_4_NaO_4_ [M + Na]^+^, 353.0468; found, 353.0463.

4-nitro-1-(3-(trifluoromethoxy)benzyl)-1H-imidazole-2-carboxamide; **13c**

General procedure C, rt, isolated by precipitation. Benzyl bromide: 1-(bromomethyl)-3-(trifluoromethoxy)benzene (212 mg, 0.83 mol). The crude product was purified by recrystallization (EtOH) to yield yellow crystals (38 mg, 17%). Mp = 249–251 °C (decomposed). LCMS: R_t_ = 3.45 min, 99 A% @ 254 nm, [M + H]^+^ = 331.1. ^1^H NMR (600 MHz, DMSO-*d*_6_) δ 8.79 (s, 1H), 8.23 (s, 1H), 7.87 (s, 1H), 7.50 (t, *J* = 8.0 Hz, 1H), 7.40 (br s, 1H), 7.35 – 7.32 (m, 2H), 5.76 (s, 2H). ^13^C NMR (150 MHz, DMSO-*d*_6_) δ 159.2, 148.3 (q, *J* = 2.2 Hz), 144.8, 139.1, 136.9, 130.6, 126.6, 125.6, 120.4, 120.2, 119.9 (q, *J* = 257.3 Hz), 50.7.. HRMS (ESI): *m/z* calcd for C_12_H_9_F_3_N_4_NaO_4_ [M + Na]^+^, 353.0468; found, 353.0480.

1-(4-methylphenethyl)-4-nitro-1H-imidazole-2-carboxamide; **13d**

General procedure C, heated in the microwave at 80 °C for 15 min then heated in the microwave at 120 °C for 15 min, isolated by aqueous work up. Alkyl halide: 4-methylphenethyl bromide (117 μL, 0.76 mmol). The reaction was heated at 80 °C for 30 mins. The crude material was purified by MPLC over silica gel (Biotage, 12–100% EtOAc in pet. spirits gradient) to yield a yellow powder (67 mg, 42%). LCMS: R_t_ = 3.38 min, 97 A% @ 254 nm, [M + H]^+^ = 274.9. ^1^H NMR (600 MHz, DMSO-*d*_6_) δ 8.49 (s, 1H), 8.15 (s, 1H), 7.82 (s, 1H), 7.11 – 7.10 (m, 2H), 7.08 – 7.07 (m, 2H), 4.68 – 4.66 (m, 2H), 3.06 – 3.03 (m, 2H), 2.26 (s, 3H). ^13^C NMR (150 MHz, DMSO-*d*_6_) δ 159.4, 144.3, 137.2, 135.6, 134.3, 129.1, 128.6, 125.6, 49.9, 36.0, 20.7. HRMS (ESI): *m/z* calcd for C_13_H_14_N_4_NaO_3_ [M + Na]^+^, 297.0958; found, 297.0961.

4-nitro-1-(pyridin-2-ylmethyl)-1H-imidazole-2-carboxamide; **13e**

General procedure C, rt, isolated by precipitation. Benzyl bromide: 2-(bromomethyl)pyridine (195 mg, 1.1 mmol). The filtered solid was washed with ice cold water and dried *in vacuo* to yield an off-white crystalline solid (97 mg, 53%). LCMS: R_t_ = 2.66 min, 99 A% @ 254 nm, [M + H]^+^ = 248.1. ^1^H NMR (600 MHz, DMSO-*d*_6_) δ 8.67 (s, 1H), 8.46 (ddd, *J* = 4.8, 1.8, 1 Hz, 1H), 8.16 (s, 1H), 7.80 (td, *J* = 7.7, 1.8 Hz, 1H), 7.73 (s, 1H), 7.30 (ddd, *J* = 7.5, 4.8, 1.3 Hz, 1H), 7.29 (dt, *J* = 7.9, 1.0 Hz, 1H), 5.85 (s, 2H). ^13^C NMR (150 MHz, DMSO-*d*_6_) δ 159.3, 155.2, 149.1, 144.5, 137.6, 137.0, 126.6, 122.8, 122.8, 52.8.. HRMS (ESI): *m/z* calcd for C_10_H_9_N_5_NaO_3_ [M + Na]^+^, 270.0598; found, 270.0593.

1-(cyclohexylmethyl)-4-nitro-1H-imidazole-2-carboxamide; **13f**

**

General procedure C. (Bromomethyl)cyclohexane (200 μL, 1.4 mmol), 60 °C for 3 days, then an additional portion of (bromomethyl)cyclohexane (200 μL, 1.4 mmol) was added and the reaction was heated at 60 °C for a further 24 hr. Isolated by aqueous work up. The crude material was purified by MPLC over silica gel (Biotage, 6–50% EtOAc in pet. spirits gradient) to yield a cream solid (70 mg, 32%). LCMS: R_t_ = 3.38 min, 99 A% @ 254 nm, [M + H]^+^ = 253.1. ^1^H NMR (600 MHz, DMSO-*d*_6_) δ 8.58 (s, 1H), 8.15 (s, 1H), 7.79 (s, 1H), 4.35 (d, *J* = 7.3 Hz, 2H), 1.81 – 1.75 (m, 1H), 1.68 – 1.66 (m, 2H), 1.61 – 1.59 (m, 2H), 1.47 (d, *J* = 12.4 Hz, 2H), 1.18 – 1.02 (m, 1H), 0.98 – 0.93 (m, 3H). ^13^C NMR (150 MHz, DMSO-*d*_6_) δ 159.5, 144.4, 137.5, 125.9, 54.0, 38.3, 29.5, 25.8, 25.1. HRMS (ESI): *m/z* calcd for C_11_H_16_N_4_NaO_3_ [M + Na]^+^, 275.1115; found, 275.1120.

1-(cyclopropylmethyl)-4-nitro-1H-imidazole-2-carboxamide; **13g**

General procedure C, heated in the microwave at 80 °C for 15 min, isolated by aqueous work up. Alkyl halide: bromomethyl cyclopropane (140 µL, 1.44 mmol). The crude material was purified by MPLC over silica gel (Biotage, 15–100% EtOAc in pet. spirits gradient) to yield a yellow solid (52 mg, 26%) LCMS: R_t_ = 2.93 min, 99 A% @ 254 nm, [M + H]^+^ = 211.1. ^1^H NMR (600 MHz, DMSO-*d*_6_) δ 8.64 (s, 1H), 8.17 (s, 1H), 7.83 (s, 1H), 4.31 (d, *J* = 7.4 Hz, 2H), 1.37 – 1.32 (m, 1H), 0.53 – 0.47 (m, 2H), 0.44 – 0.41 (m, 2H). ^13^C NMR (150 MHz, DMSO-*d*_6_) δ 159.6, 144.6, 137.1, 125.3, 53.1, 11.9, 3.6. HRMS (ESI): *m/z* calcd for C_8_H_10_N_4_NaO_3_ [M + Na]^+^, 233.0645; found, 233.0647.

N-methyl-4-nitro-1-(4-(trifluoromethoxy)benzyl)-1H-imidazole-2-carboxamide; **14a**

General procedure C, rt, isolated by aqueous workup. Benzyl bromide: 1-(bromomethyl)-4-(trifluoromethoxy)benzene (52 µL, 0.32 mmol). The crude material was purified by MPLC over silica gel (Biotage, 40–75% EtOAc in pet. spirits gradient) to yield a yellow oil (53 mg, 52%). LCMS: R_t_ = 3.55 min, 99 A% @ 254 nm, [M + H]^+^ = 345.1. ^1^H NMR (600 MHz, CDCl_3_) δ 7.77 (s, 1H), 7.41 (br s, 1H), 7.39 – 7.37 (m, 2H), 7.25 – 7.24 (m, 2H), 5.81 (s, 2H), 2.99 (d, *J* = 5.1 Hz, 3H). ^13^C NMR (150 MHz, CDCl_3_) δ 158.1, 149.5 (q, *J* = 1.8 Hz), 145.7, 136.9, 133.3, 129.8, 122.7, 121.7, 120.3 (q, *J* = 257.2 Hz), 51.9, 26.0. HRMS (ESI): *m/z* calcd for C_13_H_12_F_3_N_4_O_4_ [M + H]^+^, 345.0805; found, 345.0805.

N,N-dimethyl-4-nitro-1-(4-(trifluoromethoxy)benzyl)-1H-imidazole-2-carboxamide, **14b**

General procedure C, rt, isolated by aqueous workup. Benzyl bromide: 1-(bromomethyl)-4-(trifluoromethoxy)benzene (95 µL, 0.60 mmol). The crude material was purified by MPLC over silica gel (Biotage, 30–80% EtOAc in pet. spirits gradient) to yield a yellow oil (14 mg, 7%). LCMS: R_t_ = 3.52 min, 99 A% @ 254 nm, [M + H]^+^ = 359.1. ^1^H NMR (600 MHz, CDCl_3_) δ 7.76 (s, 1H), 7.35 – 7.34 (m, 2H), 7.26 – 7.24 (m, 2H), 5.48 (s, 2H), 3.25 (s, 3H), 3.08 (s, 3H). ^13^C NMR (150 MHz, CDCl_3_) δ 158.9, 149.5 (q, *J* = 1.8 Hz), 138.4, 133.3, 129.8, 122.9, 121.7, 121.4, 120.3 (q, *J* = 258.3 Hz), 51.6, 39.1. 35.9. HRMS (ESI): *m/z* calcd for C_14_H_14_F_3_N_4_O_4_ [M + H]^+^, 359.0962; found, 359.0959.

Ethyl 4-nitro-1-(4-(trifluoromethoxy)benzyl)-1H-imidazole-2-carboxylate; **14c**

General procedure C, rt, isolated by aqueous work up. Compound **12p** (1.0 g, 5.4 mmol), benzyl bromide: 4-(trifluoromethoxy)benzyl bromide (1.52 g, 5.96 mmol), and cesium carbonate (2.1 g, 6.48 mmol). The crude pale yellow solid was triturated several times from petroleum ether and dried under vacuum to provide the title compound as a yellow crystalline solid (1.9 g, 98%). The material was used without further purification. For characterisation and biological analysis, a small sample (200 mg) was recrystallised from EtOH (1 mL) to provide pure **14c** as a colourless solid. Mp = 112 °C. LCMS: R_t_ = 3.73 min, 97 A% @ 254 nm, [M + H]^+^ = 360.0. ^1^H NMR (600 MHz, DMSO-*d*_6_) δ 8.85 (s, 1H). 7.43 (app. d, *J* = 8.6 Hz, 2H), 7.37 (d, *J* = 8.3 Hz, 2H), 5.69 (s, 2H), 4.31 (q, *J* = 7.1 Hz, 2H), 1.26 (t, *J* = 7.1 Hz, 3H). ^13^C NMR (150 MHz, DMSO-*d*_6_) δ 157.5, 147.9 (q, *J* = 1.8 Hz), 145.6, 135.5, 134.3, 129.4, 126.3, 121.3, 120.0 (q, *J* = 256.4 Hz), 61.9, 51.2, 13.8. HRMS (ESI): *m/z* calcd for C_28_H_24_F_6_N_6_NaO_10_ [2M + Na]^+^, 741.1350; found, 741.1339.

N-hydroxy-4-nitro-1-(4-(trifluoromethoxy)benzyl)-1H-imidazole-2-carboxamide; **14d**

A stock solution of hydroxylamine was made as follows: hydroxylamine hydrochloride (1.03 g, 14.8 mmol) in HPLC grade MeOH (10 mL) was treated with a solution of KOH (0.82 g, 14.6 mmol) in MeOH (5 mL). The resulting precipitate was removed by filtration, and the clear filtrate was diluted volumetrically with MeOH to a final concentration 585 mM. Ester **14c** (102 mg, 0.28 mmol) was dissolved in the stock hydroxylamine solution (2.4 mL, 1.4 mmol) and heated to 60 °C for 18 hr. The volatiles were removed under reduced pressure, and the resulting yellow solid was purified by HPLC (Agilent, A: H_2_O + 0.1% TFA, B: ACN + 0.1% TFA, 5–100% B) to afford the title compound as a white powder (36.1 mg, 37%). LCMS: R_t_ = 3.30 min, 99 A% @ 254 nm, [M + H]^+^ = 344.9. ^1^H NMR (600 MHz, DMSO-*d*_6_) δ 11.74 (d, *J* = 1.6 Hz, 1H), 9.35 (d, *J* = 1.7 Hz, 1H), 8.80 (s, 1H), 7.48 – 7.46 (m, 2H), 7.38 – 7.37 (m, 2H), 5.72 (s, 2H). ^13^C NMR (150 MHz, DMSO-*d*_6_) δ 155.1, 147.9 (q, *J* = 1.7 Hz), 145.1, 136.0, 135.9, 129.6, 125.4, 121.3, 120.0 (q, *J* = 256.0 Hz), 50.5. HRMS (ESI): *m/z* calcd for C_24_H_18_F_6_N_8_NaO_10_ [2M + Na]^+^, 715.0942; found, 715.0920.

4-nitro-1-(4-(trifluoromethoxy)benzyl)-1H-imidazole-2-carbohydrazide; **14e**

To a stirred suspension of **14f** (50 mg, 0.15 mmol) in CH_2_Cl_2_ (10 mL) and DMF (1 µL) was added oxalyl chloride (40 µL, 0.45 mmol) at 0 °C. The mixture was warmed to rt overnight to provide a clear homogeneous solution. The volatiles were removed in vacuo, and the resulting residue was co-evaporated twice from heptane, dried in vacuo and then dissolved in anhydrous CH_2_Cl_2_ (1 mL). The resulting solution was then added dropwise via a syringe pump (flow rate 3 mL/hr) to an ice-cold, stirred solution of hydrazine monohydrate (46 µL, 0.62 mmol) in CH_2_Cl_2_ (1 mL). Once complete (determined by LCMS), the volatiles were removed in vacuo, and the resulting solid (48.7 mg) was purified by HPLC (Agilent, A: H_2_O + 0.1% TFA, B: ACN + 0.1% TFA, 5–100% B) to afford the title compound as an off-white powder (34.6 mg, 65%). LCMS: R_t_ = 3.33 min, 99 A% @ 254 nm, [M + H]^+^ = 344.0. ^1^H NMR (600 MHz, DMSO-*d*_6_) δ 8.86 (s, 1H), 7.47 (d, *J* = 8.6 Hz, 2H), 7.37 (d, *J* = 8.6 Hz, 2H), 5.73 (s, 2H). ^13^C NMR (150 MHz, DMSO*-d*_6_) δ 156.3, 148.0 (q, *J* = 1.7 Hz), 145.2, 135.8, 135.4, 129.6, 125.9, 121.3, 120.0 (q, *J* = 256.7 Hz), 50.7. HRMS (ESI): *m/z* calcd for C_12_H_11_F_3_N_5_NaO_4_ [M + Na]^+^, 346.0758; found, 346.0765.

*4-nitro-1-(4-(trifluoromethoxy)benzyl)-1H-imidazole-2-carboxylic acid****; 14f***

**

A solution of compound **14c** (1.8 g, 5.0 mmol) in THF/MeOH (1:1, 20 mL) was treated with sodium hydroxide solution (1 M, 10 mL, 10 mmol) and stirred overnight at rt. The volatiles were removed under reduced pressure to afford a yellow solution containing a fine suspension of colourless solid. The suspension was filtered through a short plug of Celite, and the resulting clear solution (~ pH 10) was washed with Et_2_O. The aqueous phase was carefully acidified with conc HCl to pH 2, resulting in formation of a precipitate accompanied by significant gas evolution. The precipitate was collected by centrifugation. The resulting pellet was re-suspended in water and then collected by vacuum filtration to afford the title compound as a colourless solid after drying under vacuum (606 mg, 37%). The aqueous phase was extracted with EtOAc to afford the corresponding decarboxylation product of the title compound (410 mg, 29%). Characterisation of **14f**: LCMS: R_t_ = 2.51 min, >97 A% @ 300 nm, [M - H]^-^ = 330.0, [M + H]^+^ = 332. ^1^H NMR (600 MHz, DMSO-*d*_6_) δ 8.65 (s, 1H), 7.48 (app. d, *J* = 8.5 Hz, 2H), 7.35 (d, *J* = 8.2 Hz, 2H), 5.77 (s, 2H). ^13^C NMR (150 MHz, DMSO-*d*_6_) δ 159.5, 147.8 (q, *J* = 1.8 Hz), 145.2, 139.9, 136.5, 129.6, 124.4, 121.2, 120.1 (q, *J* = 256.6 Hz), 50.6. HRMS (ESI): *m/z* calcd for C_12_H_8_F_3_N_3_NaO_5_ [M + Na]^+^, 354.0308; found, 354.0323.

N-(4-fluorobenzyl)-1H-imidazole-2-carboxamide; **17**

General procedure B. Amine: (4-fluorophenyl)methanamine (200 mg, 1.60 mmol), reaction solvent: anh. DCM (6 mL). After 2 hr at rt, the reaction was poured into water (60 mL) and diluted with EtOAc (40 mL). The precipitate that formed between the two layers was collected by filtration. The precipitate was washed with water (2 mL) and pet. spirits (2 mL) and dried in vacuo to yield a tan solid (236 mg, 67%). LCMS: R_t_ = 2.37 min, 99 A% @ 254 nm, [M + H]^+^ = 219.8. ^1^H NMR (600 MHz, DMSO-*d*_6_) δ 13.00 (s, 1H), 8.98 (t, *J* = 6.5 Hz, 1H), 7.36 – 7.33 (m, 2H), 7.27 (dd, *J* = 2.3, 1.1 Hz, 1H), 7.15 – 7.11 (m, 2H), 7.04 (t, *J* = 1.3 Hz, 1H), 4.40 (d, *J* = 6.4 Hz, 2H). ^13^C NMR (150 MHz, DMSO-*d*_6_) δ 161.1 (d, *J* = 243 Hz), 158.4, 140.9, 135.8, 129.3 (d, *J* = 8.3 Hz), 128.8, 119.8, 114.9 (d, *J* = 21.4 Hz), 41.2. HRMS (ESI): *m/z* calcd for C_11_H_11_FN_3_O [M + H]^+^, 220.0881; found, 220.0878.

1-(4-(trifluoromethoxy)benzyl)-1H-imidazole-2-carboxamide; **18**

General procedure C, rt, isolated by precipitation. Imidazole **19** (100 mg, 0.90 mmol), benzyl bromide: 1-(bromomethyl)-4-(trifluoromethoxy)benzene (174 µL, 1.08 mmol, 1.2 eq). The crude material was purified by MPLC over silica gel (Biotage, 50–100% EtOAc in pet. spirits gradient) to yield a colourless solid (150 mg, 58 %). LCMS: R_t_ = 3.28 min, 99 A% @ 254 nm, [M + H]^+^ = 286.1. ^1^H NMR (600 MHz, DMSO-*d*_6_) δ 7.84 (br s, 1H), 7.51 (d, *J* = 1.1 Hz, 1H), 7.48 (br s, 1H), 7.35 (s, 4H), 7.05 (d, *J* = 1.1 Hz, 1H), 5.72 (s, 2H). ^13^C NMR (150 MHz, DMSO-*d_6_*) δ 160.7, 147.5 (q, *J* = 1.8 Hz), 138.1, 137.5, 129.0, 127.6, 125.2, 121.1, 120.0 (q, *J* = 256.4 Hz), 49.2. HRMS (ESI): *m/z* calcd for C_12_H_11_F_3_N_3_O_2_ [M + H]^+^, 286.0798; found, 286.0800.

1H-imidazole-2-carboxamide; **19**

To a stirred suspension of 1H-imidazole-2-carboxylic acid (3.00 g, 26.8 mmol) in anh. DCM (45 mL) cooled to 0 °C under N_2_ was added oxalyl chloride (4.5 mL, 53.5 mmol) followed by addition of DMF (2 drops). The reaction was allowed to warm to rt and stir overnight. A further portion of oxalyl chloride (1.13 mL, 13.4 mmol) and DMF (1 drop) was added and the reaction stirred for 4 hr. Volatiles were then removed *in vacuo* and the residual oxalyl chloride was removed by co-evaporation with toluene to afford acid chloride as a colourless solid. The solid was then added portionwise to conc NH_4_OH (15 mL) chilled to 0 °C. Volatiles were then removed *in vacuo*. Residual volatiles were coevaporated with toluene to obtain dry material (25: 75 starting material:product). The crude was retreated to the reaction with oxalyl chloride (1.7 mL, 20.1 mmol) using the above procedure. The solid obtained was then washed with water (2 x 20 mL) and dried in vacuo to obtain a tan solid (2.16 g, 73%) that was used without further purification. LCMS (Waters Atlantis: Gradient Method 2): R_t_ = 1.44 min, 99 A% @ 254 nm, [M + H]^+^ = 112.2. ^1^H NMR (600 MHz, DMSO-*d*_6_) δ 12.95 (s, 1H), 7.73 (s, 4H), 7.43 (s, 1H), 7.23 (br s, 1H), 7.04 (br s, 1H). Literature reference [5].

4-amino-N-(4-fluorobenzyl)-1H-imidazole-2-carboxamide; **20**

A solution of nitroimidazole (50 mg, 189 µmol) in MeOH (6 mL) was reduced to the amine product by hydrogenation (ThalesNano H-Cube Pro, 10% Pd/C 30 mm cartridge, 1 mL/min, 1 atm, 30 °C). The eluent was evaporated to afford the crude product. Purification by MPLC over C18 silica gel (Grace Reveleris X2, A: H_2_O + 0.1% TFA, B: ACN + 0.1% TFA, 5-30% B) yielded a pink solid (33 mg, 74%). LCMS: R_t_ = 2.47 min, 99 A% @ 254 nm, [M + H]^+^ = 235.1. ^1^H NMR (600 MHz, CD_3_CN) δ 9.19 (s, 1H), 7.37 – 7.34 (m, 2H), 7.08 – 7.04 (m, 2H), 6.51 (s, 1H), 4.49 (d, *J* = 6.3 Hz, 2H). ^13^C NMR (150 MHz, CD_3_CN) δ 162.9 (d, J = 243.2 Hz), 154.9, 142.8, 135.4 (d, *J* = 3.6 Hz), 132.3, 130.5 (d, *J* = 8.2 Hz), 116.1 (d, *J* = 21.4 Hz), 101.6, 43.2. HRMS (ESI): *m/z* calcd for C_11_H_12_FN_4_O [M + H]^+^, 235.0990; found, 235.0991.

## Spectra

**Potassium 1-methyl-5-nitro-1H-imidazole-2-carboxylate; 6**

^1^H NMR (600 MHz, DMSO-*d*_6_)

**N-(4-fluorobenzyl)-1-methyl-5-nitro-1H-imidazole-2-carboxamide; 8a**

^1^H NMR (600 MHz, DMSO-*d_6_*)

**N-(4-fluorobenzyl)-1-methyl-5-nitro-1H-imidazole-2-carboxamide; 8a**

^13^C NMR (150 MHz, DMSO-*d_6_*)

1-methyl-5-nitro-N-(4-(trifluoromethoxy)benzyl)-1H-imidazole-2-carboxamide; **8b**

^1^H NMR (600 MHz, DMSO-*d*_6_)

1-methyl-5-nitro-N-(4-(trifluoromethoxy)benzyl)-1H-imidazole-2-carboxamide; **8b**

^13^C NMR (150 MHz, DMSO-*d*_6_)

1-methyl-5-nitro-N-(3-(trifluoromethoxy)benzyl)-1H-imidazole-2-carboxamide; **8c**

^1^H NMR (600 MHz, DMSO-*d_6_*)

**

**

1-methyl-5-nitro-N-(3-(trifluoromethoxy)benzyl)-1H-imidazole-2-carboxamide; **8c**

^13^C NMR (150 MHz, DMSO-*d_6_*)

**

N-(1-(4-fluorophenyl)ethyl)-1-methyl-5-nitro-1H-imidazole-2-carboxamide; **8d**

^1^H NMR (600 MHz, DMSO-*d*_6_)

N-(1-(4-fluorophenyl)ethyl)-1-methyl-5-nitro-1H-imidazole-2-carboxamide; **8d**

^13^C NMR (150 MHz, DMSO-*d*_6_)

1-methyl-N-(4-methylphenethyl)-5-nitro-1H-imidazole-2-carboxamide; **8e**

^1^H NMR (600 MHz, CDCl_3_)

1-methyl-N-(4-methylphenethyl)-5-nitro-1H-imidazole-2-carboxamide; **8e**

^13^C JMOD NMR (150 MHz, CDCl_3_)

******

1-methyl-5-nitro-N-(pyridin-2-ylmethyl)-1H-imidazole-2-carboxamide.xTFA; **8f**

^1^H NMR (600 MHz, DMSO-*d_6_*)

1-methyl-5-nitro-N-(pyridin-2-ylmethyl)-1H-imidazole-2-carboxamide.xTFA; **8f**

^13^C JMOD NMR (150 MHz, DMSO-*d_6_*)

N,N,1-trimethyl-5-nitro-1H-imidazole-2-carboxamide; **8g**

^1^H NMR (600 MHz, CDCl_3_)

N,N,1-trimethyl-5-nitro-1H-imidazole-2-carboxamide; **8g**

^13^C JMOD NMR (150 MHz, CDCl_3_)

(1-methyl-5-nitro-1H-imidazol-2-yl)(morpholino)methanone; **8h**

^1^H NMR (600 MHz, CDCl_3_)

**

(1-methyl-5-nitro-1H-imidazol-2-yl)(morpholino)methanone; **8h**

^13^C JMOD NMR (150 MHz, CDCl_3_)

(1-methyl-5-nitro-1H-imidazol-2-yl)(pyrrolidin-1-yl)methanone; **8i**

^1^H NMR (600 MHz, CDCl_3_)

(1-methyl-5-nitro-1H-imidazol-2-yl)(pyrrolidin-1-yl)methanone; **8i**

^13^C NMR (150 MHz, CDCl_3_)

N-cyclopropyl-1-methyl-5-nitro-1H-imidazole-2-carboxamide; **8j**

^1^H NMR (600 MHz, CDCl_3_)

**

**

N-cyclopropyl-1-methyl-5-nitro-1H-imidazole-2-carboxamide; **8j**

^13^C NMR (150 MHz, DMSO-*d_6_*)

**

N-cyclohexyl-1-methyl-5-nitro-1H-imidazole-2-carboxamide; **8k**

^1^H NMR (600 MHz, CDCl_3_)

N-cyclohexyl-1-methyl-5-nitro-1H-imidazole-2-carboxamide; **8k**

^13^C JMOD NMR (150 MHz, CDCl_3_)

5-nitro-1H-imidazole-2-carboxylic acid; **10**

^1^H NMR (400 MHz, DMSO-*d_6_*)

5-nitro-1H-imidazole-2-carboxylic acid; **10**

^13^C JMOD NMR (150 MHz, DMSO-*d_6_*)

N-(4-fluorobenzyl)-5-nitro-1H-imidazole-2-carboxamide; **12a**

^1^H NMR (600 MHz, DMSO-*d_6_*)

N-(4-fluorobenzyl)-5-nitro-1H-imidazole-2-carboxamide; **12a**

^13^C JMOD NMR (150 MHz, DMSO-*d_6_*)

5-nitro-N-(4-(trifluoromethoxy)benzyl)-1H-imidazole-2-carboxamide; **12b**

^1^H NMR (600 MHz, DMSO-*d_6_*)

5-nitro-N-(4-(trifluoromethoxy)benzyl)-1H-imidazole-2-carboxamide; **12b**

^13^C NMR (150 MHz, DMSO-*d_6_*)

1-methyl-5-nitro-N-(3-(trifluoromethoxy)benzyl)-1H-imidazole-2-carboxamide; **12c**

^1^H NMR (150 MHz, DMSO-*d_6_*)

1-methyl-5-nitro-N-(3-(trifluoromethoxy)benzyl)-1H-imidazole-2-carboxamide; **12c**

^13^C JMOD NMR (150 MHz, DMSO-*d_6_*)

N-(1-(4-fluorophenyl)ethyl)-5-nitro-1H-imidazole-2-carboxamide; **12d**

^1^H NMR (600 MHz, DMSO-*d_6_*)

N-(1-(4-fluorophenyl)ethyl)-5-nitro-1H-imidazole-2-carboxamide; **12d**

^13^C JMOD NMR (150 MHz, DMSO-*d_6_*)

N-(4-methylphenethyl)-5-nitro-1H-imidazole-2-carboxamide; **12e**

^1^H NMR (600 MHz, DMSO-*d_6_*)

N-(4-methylphenethyl)-5-nitro-1H-imidazole-2-carboxamide; **12e**

^13^C JMOD NMR (150 MHz, DMSO-*d_6_*)

5-nitro-N-(pyridin-2-ylmethyl)-1H-imidazole-2-carboxamide; **12f**

^1^H NMR (600 MHz, DMSO-*d_6_*)

5-nitro-N-(pyridin-2-ylmethyl)-1H-imidazole-2-carboxamide; **12f**

^13^C JMOD NMR (150 MHz, DMSO-*d_6_*)

N,N-dimethyl-5-nitro-1H-imidazole-2-carboxamide; **12g**

^1^H NMR (600 MHz, DMSO-*d_6_*)

N,N-dimethyl-5-nitro-1H-imidazole-2-carboxamide; **12g**

^13^C JMOD NMR (150 MHz, DMSO-*d_6_*)

Morpholino(5-nitro-1H-imidazol-2-yl)methanone; **12h**

^1^H NMR (600 MHz, DMSO-*d_6_*)

Morpholino(5-nitro-1H-imidazol-2-yl)methanone; **12h**

^13^C JMOD NMR (150 MHz, DMSO-*d_6_*)

(5-nitro-1H-imidazol-2-yl)(pyrrolidin-1-yl)methanone; **12i**

^1^H NMR (600 MHz, DMSO-*d_6_*)

(5-nitro-1H-imidazol-2-yl)(pyrrolidin-1-yl)methanone; **12i**

^13^C JMOD NMR (150 MHz, DMSO-*d_6_*)

N-cyclopropyl-5-nitro-1H-imidazole-2-carboxamide; **12j**

^1^H NMR (600 MHz, DMSO-*d_6_*)

N-cyclopropyl-5-nitro-1H-imidazole-2-carboxamide; **12j**

^13^C JMOD NMR (150 MHz, DMSO-*d_6_*)

N-cyclohexyl-5-nitro-1H-imidazole-2-carboxamide; **12k**

^1^H NMR (600 MHz, DMSO-*d_6_*)

N-cyclohexyl-5-nitro-1H-imidazole-2-carboxamide; **12k**

^13^C JMOD NMR (150 MHz, DMSO-*d_6_*)

5-nitro-1H-imidazole-2-carboxamide; **12l**

^1^H NMR (600 MHz, DMSO-*d_6_*)

5-nitro-1H-imidazole-2-carboxamide; **12l**

^13^C JMOD NMR (150 MHz, DMSO-*d_6_*)

N-methyl-5-nitro-1H-imidazole-2-carboxamide; **12m**

^1^H NMR (600 MHz, DMSO-*d_6_*)

N-methyl-5-nitro-1H-imidazole-2-carboxamide; **12m**

^13^C JMOD NMR (150 MHz, DMSO-*d_6_*)

N-(2-hydroxyethyl)-5-nitro-1H-imidazole-2-carboxamide; **12n**

^1^H NMR (600 MHz, DMSO-*d_6_*)

Coincident methylene and water proton resonances precluded accurate integration of methylene protons at 3.33 pm.

N-(2-hydroxyethyl)-5-nitro-1H-imidazole-2-carboxamide; **12n**

^13^C JMOD NMR (150 MHz, DMSO-*d_6_*)

N-(2-hydroxyethyl)-N-methyl-5-nitro-1H-imidazole-2-carboxamide; **12o**

^1^H NMR (600 MHz, DMSO-*d_6_*)

N-(2-hydroxyethyl)-N-methyl-5-nitro-1H-imidazole-2-carboxamide; **12o**

^13^C JMOD NMR (150 MHz, DMSO-*d_6_*)

Ethyl 5-nitro-1H-imidazole-2-carboxylate; **12p**

^1^H NMR (600 MHz, DMSO-*d_6_*)

Ethyl 5-nitro-1H-imidazole-2-carboxylate; **12p**

^13^C JMOD NMR (150 MHz, DMSO-*d_6_*)

1-(4-fluorobenzyl)-4-nitro-1H-imidazole-2-carboxamide; **13a**

^1^H NMR (600 MHz, DMSO-*d_6_*)

1-(4-fluorobenzyl)-4-nitro-1H-imidazole-2-carboxamide; **13a**

^13^C JMOD NMR (150 MHz, DMSO-*d_6_*)

4-nitro-1-(4-(trifluoromethoxy)benzyl)-1H-imidazole-2-carboxamide; **13b**

^1^H NMR (600 MHz, DMSO-*d_6_*)

4-nitro-1-(4-(trifluoromethoxy)benzyl)-1H-imidazole-2-carboxamide; **13b**

^13^C JMOD NMR (150 MHz, DMSO-*d_6_*)

4-nitro-1-(3-(trifluoromethoxy)benzyl)-1H-imidazole-2-carboxamide; **13c**

^1^H NMR (600 MHz, DMSO-*d_6_*)

4-nitro-1-(3-(trifluoromethoxy)benzyl)-1H-imidazole-2-carboxamide; **13c**

^13^C JMOD NMR (150 MHz, DMSO-*d_6_*)

1-(4-methylphenethyl)-4-nitro-1H-imidazole-2-carboxamide; **13d**

^1^H NMR (600 MHz, DMSO-*d_6_*)

1-(4-methylphenethyl)-4-nitro-1H-imidazole-2-carboxamide; **13d**

^13^C JMOD NMR (150 MHz, DMSO-*d_6_*)

4-nitro-1-(pyridin-2-ylmethyl)-1H-imidazole-2-carboxamide; **13e**

^1^H NMR (600 MHz, DMSO-*d_6_*)

4-nitro-1-(pyridin-2-ylmethyl)-1H-imidazole-2-carboxamide; **13e**

^13^C JMOD NMR (150 MHz, DMSO-*d_6_*)

1-(cyclohexylmethyl)-4-nitro-1H-imidazole-2-carboxamide; **13f**

^1^H NMR (600 MHz, DMSO-*d_6_*)

1-(cyclohexylmethyl)-4-nitro-1H-imidazole-2-carboxamide; **13f**

^13^C JMOD NMR (150 MHz, DMSO-*d_6_*)

1-(cyclopropylmethyl)-4-nitro-1H-imidazole-2-carboxamide; **13g**

^1^H NMR (600 MHz, DMSO-*d_6_*)

1-(cyclopropylmethyl)-4-nitro-1H-imidazole-2-carboxamide; **13g**

^13^C JMOD NMR (150 MHz, DMSO-*d_6_*)

N-methyl-4-nitro-1-(4-(trifluoromethoxy)benzyl)-1H-imidazole-2-carboxamide; **14a**

^1^H NMR (600 MHz, CDCl_3_)

N-methyl-4-nitro-1-(4-(trifluoromethoxy)benzyl)-1H-imidazole-2-carboxamide; **14a**

^13^C JMOD NMR (150 MHz, CDCl_3_)

N,N-dimethyl-4-nitro-1-(4-(trifluoromethoxy)benzyl)-1H-imidazole-2-carboxamide, **14b**

^1^H NMR (600 MHz, CDCl_3_)

N,N-dimethyl-4-nitro-1-(4-(trifluoromethoxy)benzyl)-1H-imidazole-2-carboxamide, **14b**

^13^C JMOD NMR (150 MHz, CDCl_3_)

Ethyl 4-nitro-1-(4-(trifluoromethoxy)benzyl)-1H-imidazole-2-carboxylate; **14c**

^1^H NMR (600 MHz, DMSO-*d_6_*)

Ethyl 4-nitro-1-(4-(trifluoromethoxy)benzyl)-1H-imidazole-2-carboxylate; **14c**

^13^C JMOD NMR (150 MHz, DMSO-*d_6_*)

N-hydroxy-4-nitro-1-(4-(trifluoromethoxy)benzyl)-1H-imidazole-2-carboxamide; **14d**

^1^H NMR (600 MHz, DMSO-*d_6_*)

N-hydroxy-4-nitro-1-(4-(trifluoromethoxy)benzyl)-1H-imidazole-2-carboxamide; **14d**

^13^C JMOD NMR (150 MHz, DMSO-*d_6_*)

4-nitro-1-(4-(trifluoromethoxy)benzyl)-1H-imidazole-2-carbohydrazide; **14e**

^1^H NMR (600 MHz, DMSO-*d_6_*)

4-nitro-1-(4-(trifluoromethoxy)benzyl)-1H-imidazole-2-carbohydrazide; **14e**

^13^C JMOD NMR (150 MHz, DMSO-*d_6_*)

*4-nitro-1-(4-(trifluoromethoxy)benzyl)-1H-imidazole-2-carboxylic acid;* ***14f***

^1^H NMR (600 MHz, DMSO-*d_6_*)

*4-nitro-1-(4-(trifluoromethoxy)benzyl)-1H-imidazole-2-carboxylic acid;* ***14f***

^13^C JMOD NMR (150 MHz, DMSO-*d_6_*)

N-(4-fluorobenzyl)-1H-imidazole-2-carboxamide; **17**

^1^H NMR (600 MHz, DMSO-*d_6_*)

N-(4-fluorobenzyl)-1H-imidazole-2-carboxamide; **17**

^13^C JMOD NMR (150 MHz, DMSO-*d_6_*)

1-(4-(trifluoromethoxy)benzyl)-1H-imidazole-2-carboxamide; **18**

^1^H NMR (600 MHz, DMSO-*d_6_*)

1-(4-(trifluoromethoxy)benzyl)-1H-imidazole-2-carboxamide; **18**

^13^C NMR (150 MHz, DMSO-*d_6_*)

1H-imidazole-2-carboxamide; **19**

^1^H NMR (600 MHz, DMSO-*d_6_*)

4-Amino-N-(4-fluorobenzyl)-1H-imidazole-2-carboxamide; **20**

^1^H NMR (600 MHz, CD_3_CN)

4-Amino-N-(4-fluorobenzyl)-1H-imidazole-2-carboxamide; **20**

^13^C JMOD NMR (150 MHz, CD_3_CN)

## Supplementary Methods

### LC/MS/MS detection and analysis parameters for plasma protein binding and metabolic stability

LC/MS/MS analysis was performed using AB Sciex 4000 QTRAP System and Shimadzu Nexara UPLC System equipped with Waters Atlantis T3, 2.1 × 50 mm, 5 µm, with guard column and column temperature was set to 40 °C. Injection volume was 5 μL and the autosampler cooler was set to 12 °C. The flow rate was 0.2 mL/min with gradient: 5% B for 1 minute then 5–100% B for 5 minutes (A: 0.1% (v/v) formic Acid in water and B: 0.1% (v/v) formic acid in Acetonitrile). Mass spectrometer parameters in Selected Reaction Monitoring (SRM) mode are tabulated below:

Table S1. MS parameters for Selected Reaction Monitoring (SRM).

| Compound | X | R | Ionization  Mode | Declustering  Potential | Collision  Energy | *m/z* |
| --- | --- | --- | --- | --- | --- | --- |
| **sulfamethoxazole** |  | | positive | 60 | 17 | 254.30→155.9 |
| **verapamil** |  | | positive | 70 | 35 | 455.3→165.3 455.3→303.3 |
| **carbutamide** |  | | positive | 70 | 25 | 272.1→155.8 |
| **carbutamide** |  | | negative | -70 | -25 | 270.2→170.8 |
| **1** | **Metronidazole** | | positive | 70 | 25 | 172.0→127.8 172.0→81.9 |
| **8a** | Me | NHCH_2_(4-F-Ph) | positive | 70 | 20 | 279.0→108.7 |
| **8h** | Me | morpholine | positive | 70 | 33 | 241.0→171.8 241.0→113.9  241.0→69.9 |
| **8k** | Me | NH-cyclohexyl | positive | 70 | 25 | 252.8→170.8 252.8→83.0 |
| **12a** | H | NHCH_2_(4-F-Ph) | negative | -70 | -35 | 262.8→64.8 262.8→111.8 |
| **12h** | H | morpholine | negative | -70 | -40 | 224.9→111.1 224.9→94.9  224.9→64.8 |
| **12k** | H | NH-cyclohexyl | negative | -90 | -38 | 236.8→64.8 236.8→111.8 |

## Supplementary Results

### Correlation of compound activity and properties

Table S2: Correlation analysis of compound activity and properties.

|  | *E. histolytica* | *G. lamblia* | *T. vaginalis* | *C. difficile* | MW | ALogP | LogD | tPSA | logS |
| --- | --- | --- | --- | --- | --- | --- | --- | --- | --- |
| *E. histolytica* | 1.00 |  |  |  |  |  |  |  |  |
| *G. lamblia* | 0.56 | 1.00 |  |  |  |  |  |  |  |
| *T. vaginalis* | 0.84 | 0.67 | 1.00 |  |  |  |  |  |  |
| *C. difficile* | -0.61 | -0.88 | -0.65 | 1.00 |  |  |  |  |  |
| MW | 0.39 | 0.82 | 0.50 | -0.75 | 1.00 |  |  |  |  |
| ALogP | 0.48 | 0.94 | 0.53 | -0.72 | 0.91 | 1.00 |  |  |  |
| LogD | 0.47 | 0.93 | 0.53 | -0.72 | 0.91 | 1.00 | 1.00 |  |  |
| tPSA | -0.62 | 0.06 | -0.26 | -0.23 | 0.09 | -0.09 | -0.08 | 1.00 |  |
| LogS | -0.40 | -0.92 | -0.49 | 0.72 | -0.92 | -0.97 | -0.97 | -0.04 | 1.00 |

Figure S1. Activity vs logD for each organism with linear regression analysis. Metronidazole (black symbols) is shown for comparison.

Figure S2. Activity vs MW for each organism with linear regression analysis. Metronidazole (black symbols) is shown for comparison.

Figure S3. Activity vs logS for each organism with linear regression analysis. Metronidazole (black symbols) is shown for comparison.

### Activity of nitroimidazole carboxamides against *C. difficile*

Table S3. Activity of 1-methyl 5-nitroimidazole and 1*H*-4(5)-nitroimidazole carboxamides against *C. difficile*.

|  | | | MIC (µg/mL) | |
| --- | --- | --- | --- | --- |
|  |  |  | *C. difficile* | |
|  |  |  | 630 | NAP1/027 |
| **Compound** | **X** | **R** |  |  |
| **1** | **metronidazole** | | 0.5 | 0.5 |
| **8a** | Me | NHCH_2_(4-F-Ph) | >64 | >64 |
| **8b** | Me | NHCH_2_(4-OCF_3_-Ph) | 32 | 32 |
| **8c** | Me | NHCH_2_(3-OCF_3_-Ph) | 32-64 | 64 |
| **8d** | Me | NHCHMe(4-F-Ph) | 64 | >64 |
| **8e** | Me | NHCH_2_CH_2_(4-Me-Ph) | >64 | >64 |
| **8f** | Me | NHCH_2_(2-pyridinyl) | 64 | 64 |
| **8g** | Me | N(Me)_2_ | 32 | 64 |
| **8h** | Me | morpholine | 32 | 32 |
| **8i** | Me | pyrrolidine | 64 | 64 |
| **8j** | Me | NH-cyclopropyl | >64 | >64 |
| **8k** | Me | NH-cyclohexyl | >64 | >64 |
|  |  |  |  |  |
| **12a** | H | NHCH_2_(4-F-Ph) | 4 | 4 |
| **12b** | H | NHCH_2_(4-OCF_3_-Ph) | 16 | 8-16 |
| **12c** | H | NHCH_2_(3-OCF_3_-Ph) | 16 | 16 |
| **12d** | H | NHCHMe(4-F-Ph) | 8 | 8 |
| **12e** | H | NHCH_2_CH_2_(4-Me-Ph) | 8 | 8 |
| **12f** | H | NHCH_2_(2-pyridinyl) | 2 | 2 |
| **12g** | H | N(Me)_2_ | 2 | 2 |
| **12h** | H | morpholine | 2 | 2 |
| **12i** | H | pyrrolidine | 2 | 2 |
| **12j** | H | NH-cyclopropyl | 1 | 1 |
| **12k** | H | NH-cyclohexyl | 8 | 8 |
| **12l** | H | NH_2_ | 1 | 1-2 |
| **12m** | H | NHMe | 0.5-1 | 1 |
| **12n** | H | NHCH_2_CH_2_OH | 8 | 8-16 |
| **12o** | H | NHCHMeCH_2_OH | 2 | 4 |

Table S4. Activity of 1-substituted 4-nitroimidazoles against *C. difficile*.

|  | | | MIC (µg/mL) | |
| --- | --- | --- | --- | --- |
|  |  |  | *C. difficile* | |
| **Compound** | **X** | **R** | 630 | NAP1/027 |
| **1** | **metronidazole** | | 0.5 | 0.5 |
| **13d** | CH_2_(4-F-Ph) | -NH_2_ | >64 | >64 |
| **13c** | CH_2_(4-OCF_3_-Ph) | -NH_2_ | >64 | N.D |
| **13f** | CH_2_(3-OCF_3_-Ph) | -NH_2_ | >64 | >64 |
| **13g** | CH_2_CH_2_(4-Me-Ph) | -NH_2_ | >64 | >64 |
| **13i** | CH_2_(2-pyridinyl) | -NH_2_ | >64 | >64 |
| **13k** | CH_2_-cyclopropyl | -NH_2_ | >64 | >64 |
| **13j** | CH_2_-cyclohexyl | -NH_2_ | >64 | >64 |
|  |  |  |  |  |
| **14a** | CH_2_(4-OCF_3_-Ph) | -NHMe | 64->64 | >64 |
| **14b** | CH_2_(4-OCF_3_-Ph) | -NMe_2_ | 64 | 64 |
| **14c** | CH_2_(4-OCF_3_-Ph) | -OEt | >64 | >64 |
| **14d** | CH_2_(4-OCF_3_-Ph) | -NHOH | 16 | 16 |
| **14e** | CH_2_(4-OCF_3_-Ph) | -NHNH_2_ | >64 | >64 |

### Desnitro and amine derivatives were inactive against *G. lamblia*, *E. histolytica*, *T. vaginalis* and *C. difficile*.

Table S5. Activity of desnitro-imidazole derivatives 17 and 18 and amine-imidazole 20 against *G. lamblia*, *E. histolytica*, *T. vaginalis* and *C. difficile*.

|  | | EC_50_ (µM) | | | MIC (µg/mL) |
| --- | --- | --- | --- | --- | --- |
|  |  | *G. lamblia* | *E. histolytica* | *T. vaginalis* | *C. difficile* |
| **Compound** |  | WB | HM1:IMSS | F1623 | 630 |
| **1** | **Metronidazole** | 6.1 | 5.0 | 0.8 | 0.5 |
| **17** |  | >50 | >50 | >20 | >64 |
| **18** |  | >50 | >50 | N.D | >64 |
| **20** |  | >50 | >50 | >20 | >64 |

## References

[1] L. Dreier, G. Wider, Concentration measurements by PULCON using X-filtered or 2D NMR spectra, Magn Reson Chem. 44 Spec No (2006) S206–12. doi:10.1002/mrc.1838.

[2] D.R. Hoff, Nitroimidazole carboxamides, US Patent 3715364, 1973.

[3] O. Marder, F. Albericio, Industrial application of coupling reagents in peptides, Chim. Oggi. 6 (2003) 35–40.

[4] H. Zimmerman, E. Brueckener, K. Henninger, U. Rosentreter, M. Hendrix, J. Kelenich, et al., eds., Heterocyclyamide-substituted imidazoles, US 2008/0176859 A1, 2008.

[5] J.P. Dirlam, R.B. James, E.V. Shoop, Syntheses and reactions of some 4,5-dihaloimidazole-2-carboxylic acid derivatives, Journal of Heterocyclic Chemistry. 17 (1980) 409–411. doi:10.1002/jhet.5570170244.
